# Different whole-brain functional connectivity correlates of reactive-proactive aggression and callous-unemotional traits in children and adolescents with disruptive behaviors

**DOI:** 10.1016/j.nicl.2023.103542

**Published:** 2023-11-13

**Authors:** Julia E. Werhahn, Lukasz Smigielski, Seda Sacu, Susanna Mohl, David Willinger, Jilly Naaijen, Leandra M. Mulder, Jeffrey C. Glennon, Pieter J. Hoekstra, Andrea Dietrich, Renee Kleine Deters, Pascal M. Aggensteiner, Nathalie E. Holz, Sarah Baumeister, Tobias Banaschewski, Melanie C. Saam, Ulrike M.E. Schulze, David J. Lythgoe, Arjun Sethi, Michael Craig, Mathilde Mastroianni, Ilyas Sagar-Ouriaghli, Paramala J. Santosh, Mireia Rosa, Nuria Bargallo, Josefina Castro-Fornieles, Celso Arango, Maria J. Penzol, Marcel P. Zwiers, Barbara Franke, Jan K. Buitelaar, Susanne Walitza, Daniel Brandeis

**Affiliations:** a Department of Child and Adolescent Psychiatry and Psychotherapy, University Hospital of Psychiatry Zurich, University of Zurich, Zurich, Switzerland; b Department of Child and Adolescent Psychiatry and Psychotherapy, Central Institute of Mental Health, Medical Faculty Mannheim, Heidelberg University, Mannheim, Germany; c Department of Cognitive Neuroscience, Donders Institute for Brain, Cognition and Behavior, Radboud University Medical Center, Radboud University, Nijmegen, The Netherlands; d Centre for Cognitive Neuroimaging, Donders Institute for Brain, Cognition and Behavior, Radboud University, Nijmegen, The Netherlands; e Conway Institute of Biomedical and Biomolecular Research, School of Medicine, University College Dublin, Dublin, Ireland; f Department of Child and Adolescent Psychiatry, University Medical Center Groningen, University of Groningen, Groningen, The Netherlands; g Department of Child and Adolescent Psychiatry and Psychotherapy, University Hospital, University of Ulm, Ulm, Germany; h Department of Neuroimaging, Institute of Psychiatry, Psychology and Neuroscience, King’s College London, London, United Kingdom; i Department of Forensic and Neurodevelopmental Sciences, Institute of Psychiatry, Psychology and Neuroscience, King’s College London, London, United Kingdom; j Department of Child Psychiatry, Institute of Psychiatry, Psychology and Neuroscience, King’s College London, London, United Kingdom; k Child and Adolescent Psychiatry Department, Hospital Clinic of Barcelona, IDIBAPS, Barcelona, Spain; l Clinic Image Diagnostic Center (CDIC), Hospital Clinic of Barcelona, Magnetic Resonance Image Core Facility, IDIBAPS, Barcelona, Spain; m Child and Adolescent Psychiatry and Psychology Department, Institute Clinic of Neurosciences, Hospital Clinic of Barcelona, CIBERSAM, IDIBAPS, Department of Medicine, University of Barcelona, Barcelona, Spain; n Child and Adolescent Psychiatry Department, Hospital General Universitario Gregorio Marañón School of Medicine, Universidad Complutense, IiSGM, CIBERSAM, Madrid, Spain; o Departments of Human Genetics and Psychiatry, Donders Institute for Brain, Cognition and Behavior, Radboud University Medical Center. Radboud University, Nijmegen, The Netherlands; p Karakter Child and Adolescent Psychiatry University Center, Radboud University Medical Center, Radboud University, Nijmegen, The Netherlands; q Neuroscience Center Zurich, University of Zurich and ETH Zurich, Zurich, Switzerland

**Keywords:** Reactive aggression, Proactive aggression, Callous-unemotional traits, Resting-state fMRI, Children, Adolescents

## Abstract

**Background:**

Disruptive behavior in children and adolescents can manifest as reactive aggression and proactive aggression and is modulated by callous-unemotional traits and other comorbidities. Neural correlates of these aggression dimensions or subtypes and comorbid symptoms remain largely unknown. This multi-center study investigated the relationship between resting state functional connectivity (rsFC) and aggression subtypes considering comorbidities.

**Methods:**

The large sample of children and adolescents aged 8–18 years (*n* = 207; mean age = 13.30±2.60 years, 150 males) included 118 cases with disruptive behavior (80 with Oppositional Defiant Disorder and/or Conduct Disorder) and 89 controls. Attention-deficit/hyperactivity disorder (ADHD) and anxiety symptom scores were analyzed as covariates when assessing group differences and dimensional aggression effects on hypothesis-free global and local voxel-to-voxel whole-brain rsFC based on functional magnetic resonance imaging at 3 Tesla.

**Results:**

Compared to controls, the cases demonstrated altered rsFC in frontal areas, when anxiety but not ADHD symptoms were controlled for. For cases, reactive and proactive aggression scores were related to global and local rsFC in the central gyrus and precuneus, regions linked to aggression-related impairments. Callous-unemotional trait severity was correlated with ICC in the inferior and middle temporal regions implicated in empathy, emotion, and reward processing. Most observed aggression subtype-specific patterns could only be identified when ADHD and anxiety were controlled for.

**Conclusions:**

This study clarifies that hypothesis-free brain connectivity measures can disentangle distinct though overlapping dimensions of aggression in youths. Moreover, our results highlight the importance of considering comorbid symptoms to detect aggression-related rsFC alterations in youths.

## Highlights

• Disruptive behavior is associated with resting-state functional connectivity (rsFC) alterations in several brain regions implicated in emotion regulation, decision-making, and moral-related behaviors.
• Overlapping and distinct rsFC alterations were identified for aggression subtypes.
• Our findings underscored the importance of comorbid ADHD and anxiety symptoms to detect aggression-related rsFC alterations in youths.

## 1 Introduction

Severe aggressive behavior in children and adolescents is a main characteristic of DSM-5 aggression-related disorders (American Psychiatric Association, 2013). Among them, conduct disorder (CD) is defined as a persistent violation of rules, norms, and rights, including physical and psychological aggressive behaviors. Oppositional defiant disorder (ODD) refers to a pattern of angry, argumentative, and vindictive behavior. Brain studies have associated CD and ODD with frontal, striatal, and limbic functional alterations (Blair et al., 2018). Notably, the combination of childhood CD and comorbid attention-deficit/hyperactivity disorder (ADHD) is thought to be linked to persistent antisocial behavior (Frick, 2016, Moffitt, 2017, Moffitt et al., 2002). In addition to ADHD (Loeber et al., 2000), comorbid anxiety (Frick, 2012, Frick et al., 2014) also seems crucial for subtyping aggression at a psychophysiological level (Fanti, 2018).These above observations underline the relevance of considering symptomatology co-occurring with aggression and suggest that brain-related measures may mark aspects of these juvenile disorders.

Resting-state functional magnetic resonance imaging (rs-fMRI) allows for the detection of intrinsic activity and functional synchronizations in brain regions during the absence of goal-directed behaviors (i.e., task-free mental state) (Fox et al., 2015, Greicius et al., 2003, Raichle et al., 2001). This well-established approach has provided important insights into biological psychiatry in general and, specifically, in relation to aggression-related disorders. For instance, a recent coordinate-based *meta*-analysis (Dugré and Potvin, 2021) showed that individuals with antisocial behavior exhibited abnormal resting-state functional connectivity (rsFC) in frontal and parietal regions constituting the default-mode network (DMN), which confirms the findings of previous empirical work conducted in male adolescents with CD (Lu et al., 2017a, Lu et al., 2015, Zhou et al., 2016). However, altered rsFC was not only restricted to the DMN but identified also in limbic (Cao et al., 2018, Wu et al., 2017, Zhou et al., 2015), motor (Cao et al., 2018, Dugré and Potvin, 2023, Lu et al., 2021a), cerebellar (Cao et al., 2019, Cao et al., 2018, Wu et al., 2017), and visual regions (Lu et al., 2021a, Lu et al., 2017b, Lu et al., 2015, Pu et al., 2017, Zhou et al., 2016). Furthermore, these alterations encompassed both decreased (Lu et al., 2017b, Lu et al., 2017a, Zhou et al., 2016) and increased (Lu et al., 2017a, Pu et al., 2017, Uytun et al., 2017) rsFC patterns compared to controls, especially in the DMN. These studies, however, have been mostly restricted to case–control comparisons in male adolescents with CD and further limited by their small sample sizes. Research on the diverse aggression-related conditions and subclinical profiles beyond CD and ODD diagnoses is also necessary to characterize the functional brain organization underlying disruptive behavior across a spectrum.

Aggression is a heterogeneous phenotype and has frequently been subdivided into reactive aggression (RA) and proactive aggression (PA) behaviors (Dodge and Coie, 1987, Romero-Martínez et al., 2022, Vitiello and Stoff, 1997). RA is described as perceiving the behavior of others as a provocation or threat and reacting aggressively (Smeets et al., 2017), while PA is characterized by planned, instrumental aggressive behaviors. A recent systematic review of RA and PA reported morphological differences in the amygdala and temporal regions as well as higher medial prefrontal cortex activation in both aggression subtypes alongside several distinct morphological and brain activation alterations (Romero-Martínez et al., 2022). However, most of the studies they included were conducted in adults, and neural correlates of RA and PA in disruptive children and adolescents remain underexamined. To the best of our knowledge, only two previous studies have examined RA- and PA-related neural differences using functional connectivity in children and adolescents (Ibrahim et al., 2022, Werhahn et al., 2021). Ibrahim et al. (2022) employed connectome-based predictive modeling during an implicit face perception task and identified largely similar patterns of network connectivity for PA and RA in children (alongside with some distinct alterations). In our previous study, we had investigated rsFC differences related to PA and RA in children and adolescents with disruptive behavior disorder using a seed-based approach (Werhahn et al., 2021). Our results showed that both subdimensions were associated with overlapping as well as distinct effects on rsFC. Altered rsFC between the left amygdala and precuneus (see also Wong et al., 2019 for an ALE *meta*-analysis on aggression) was observed in both aggression subtypes, whereas RA was further associated with rsFC differences between parietal, temporal, occipital, and limbic regions. However, these findings were limited to the connectivity pattern of a few regions of interest selected a priori. A global approach will offer the advantage of elucidating potential RA- and PA-related rsFC differences across the whole brain

Callous-unemotional (CU) traits are thought to be important modifiers of aggression and may further shape the manifestation of aggression. These traits, characterized by limited prosocial emotions, have been added to the DSM-5 (American Psychiatric Association, 2013) to further specify CD. They were also shown to be associated with a reduced treatment response and poorer clinical outcomes (Bakker et al., 2017, Wilkinson et al., 2016). Moreover, CU traits are linked to impaired empathy (Blair et al., 2014, Ciucci et al., 2015), neurocognitive dysfunctions in emotion and reward learning processes (Reidy et al., 2017), and adult psychopathy (Frick et al., 2014, Frick and White, 2008). There is also evidence for CU traits being linked to both RA and PA (Pechorro et al., 2017) or PA alone (Urben et al., 2018). Importantly, youths with CU and CU-related traits but various levels of anxiety differ in their behavioral characteristics (Fanti et al., 2013, Kimonis et al., 2012). Prior investigations showed that male adolescents with CU traits exhibited altered amygdala sub-regional rsFC (Aghajani et al., 2016, Aghajani et al., 2017) and reduced amygdala efficiency (Jiang et al., 2021) compared to controls. Correlational studies conducted with large samples further demonstrated that CU traits were linked to rsFC abnormalities in the DMN and attention networks (Umbach and Tottenham, 2021, Werhahn et al., 2021) as well as disrupted connectivity between DMN and attention networks (Pu et al., 2017, Winters et al., 2023). However, these studies were also biased in terms of a priori selection of regions or parcellation maps. In addition, despite anxiety commonly co-occurring with prominent behavioral and psychophysiological effects (Dadds et al., 2018, Fanti et al., 2013, Guelker et al., 2014) and interacting with psychopathy levels (Motzkin et al., 2011), knowledge on rsFC correlates of CU traits is still scarce, and the impact of comorbid anxiety is unknown.

Using seed-based rsFC, we previously identified altered connectivity of the DMN and salience network regions with frontal, parietal, and occipital regions in children and adolescents with disruptive behavior disorder (Werhahn et al., 2021). In this present study, we extend our prior work by investigating unconstrained voxel-to-voxel rsFC to provide complementary and more comprehensive insights into whole-brain rsFC alterations related to disruptive behavior in this large sample of children and adolescents. Specifically, we examined the rsFC patterns in relation to RA and PA dimensions as well as CU traits, and simultaneously considered co-occurring ADHD and anxiety symptoms in the analyses. We focused on two parameters, namely global and local brain rsFC. On a global level, the intrinsic connectivity contrast (ICC) was computed to measure network centrality by calculating the global strength of connectivity for each voxel with other voxels in the rest of the brain (Martuzzi et al., 2011). This method has the advantage of circumventing the need for a priori definitions of regions of interest. ICC has been applied in prior studies (Browndyke et al., 2018, Cassady et al., 2018, Vatansever et al., 2017, Walpola et al., 2017) and has recently been combined with a measure of local coherence (Browndyke et al., 2018), i.e., integrated local correlation (ILC) (Deshpande et al., 2009). Both methods reveal complementary information on brain topology from a hemodynamic response. Based on our previous findings (Werhahn et al., 2021), we hypothesized that we would find (1) between-group differences in rsFC in frontal regions, (2) RA- and PA-related rsFC differences in the precuneus, (3) rsFC alterations of CU traits beyond (para-)limbic regions, and (4) these alterations would be partly modulated by ADHD and anxiety symptoms.

## 2 Methods and materials

### 2.1 Participants

For the present study, 118 children and adolescents (males, *n* = 99) aged 8–18 years (mean age = 13.23, *SD* = 2.68) diagnosed with ODD and/or CD and/or exhibiting clinically relevant aggression scores along with 89 healthy controls were included (Werhahn et al., 2021). Cases were recruited from resident hospitals, ambulatories, and eligible schools at nine sites in Europe within the framework of the joint EU-MATRICS and EU-Aggressotype projects. Clinically relevant aggression was defined as aggression scores in the clinical range (*T* >70) according to the Child Behavior Checklist (CBCL), Youth Self Report (YSR), or Teacher Report Form (TRF) (Achenbach, 1991). Exclusion criteria for cases were a primary DSM-diagnosis of depression, anxiety, psychosis, or bipolar disorder for cases and, for the typically developed comparison group, a DSM-diagnosis or clinically relevant aggression score in the CBCL, TRF, or YSR. Furthermore, participants were excluded in cases of contraindications for MRI scanning (i.e., braces, metal implants), insufficient language skills, or an IQ below 80 (Wechsler, 2003). Parents or legal representatives of all participants gave written informed consent. The participating sites obtained ethical approval separately from their local ethics committees. Further information on the study sample is provided in Supplementary Material S1.

### 2.2 Clinical assessments

To be included in the study, cases had to meet a DSM -diagnosis of CD and/or ODD according to the semi-structured interview Kiddie-Schedule for Affective Disorders and Schizophrenia, present and lifetime version (K-SADS-PL) (Kaufman et al., 1997) or a clinically relevant score (*T* >70) on the aggression or rule-breaking behavior subscale of the CBCL, YSR, or TRF (Achenbach, 1991). The assessment of these clinical questionnaires and the K-SADS-PL by trained (clinical) psychologists or interns also in the typically developed comparison group ensured the absence of a DSM-diagnosis or clinically relevant aggression scores. Anxiety symptoms were measured using the YSR, as internalizing problems including anxiety have been shown to exhibit higher validity (Leung et al., 2006) and reliability (Gomez et al., 2014) compared to the CBCL and TRF. The self-report Reactive-Proactive Questionnaire (RPQ) was used to measure the frequency of RA and PA symptoms (Raine et al., 2006). RA and PA symptoms within cases were strongly correlated in our sample (*r* = 0.63, *p* < 0.001), as previously reported (Cima et al., 2013, Naaijen et al., 2020). CU traits were assessed by means of the parent-reported version of the Inventory of Callous-Unemotional traits (ICU) (Essau et al., 2006), as it seems to quantify these traits better than the self-reported version (Docherty et al., 2017). To assess comorbid ADHD symptoms, parents answered the SNAP-IV (Swanson, 1992), with the inattention and hyperactivity/impulsivity subdomains being used in the present study. Four subscales of the Wechsler Intelligence Scale for Children (Block Design, Similarities, Vocabulary, Picture Completion) (Wechsler, 2003) enabled the estimation of an IQ to ensure sufficient intellectual and cognitive functioning (For more details about assessment tools and study procedure, see Supplementary Material S2).

### 2.3 Image acquisition

MRI scanning took place at nine sites across Europe, with six sites using Siemens 3T scanners, two sites using Philips 3T scanners, and one site using a GE 3T scanner. Following a T1-weighted structural scan, a T2*-weighted whole-brain echo planar imaging (EPI) resting state sequence (TR 2.45 s or less, with at least 32 slices) with an average acquisition time of 8 min 25 sec was performed (Supplementary Table S2). Participants were instructed to lie still, look at a white crosshair presented against a black background, and not think about anything in particular (Supplementary Material S2). More information on the scanning parameters is provided in the Supplementary Material S3 (Table S1 and Table S2).

### 2.4 Preprocessing

Functional magnetic resonance imaging (fMRI) data were preprocessed using SPM version 12 (https://www.fil.ion.ucl.ac.uk/spm). After realignment and unwarping (Andersson et al., 2001) and subsequent slice timing correction, multi-echo images were weighted by their echo time (TE) using MATLAB (https://www.mathworks.com, MathWorks, Natick, MA, USA). All rs-fMRI data were normalized to the Montreal Neurological Institute template brain to reduce variability across subjects (Calhoun et al., 2017) using the SPM-based functional connectivity CONN toolbox (Whitfield-Gabrieli and Nieto-Castanon, 2012) in MATLAB. Functional scans were smoothed with a Gaussian 6 mm full width at half-maximum kernel. Functional outliers exceeding three standard deviations from the mean intensity across blood-oxygen-level-dependent (BOLD) time series data and exceeding 0.5 mm composite scan-to-scan motion were identified with the Artifact Detection Toolbox (ART, https://www.nitrc.org/projects/artifact_detect) within CONN. Ten cases were excluded from further analysis, owing to their functional scans exceeding 5 % of the highest mean root mean square (RMS) framewise displacement (FD) (cut-off = 0.95 RMS-FD), the threshold applied in prior studies of adolescents with ADHD (Von Rhein et al., 2016). Moreover, 23 cases and 3 controls were further excluded owing to missing scans (*n* = 2) or insufficient quality of the structural (*n* = 10) or functional (*n* = 14) scans. In the denoising step, the aCompCor strategy (Behzadi et al., 2007) as implemented in CONN, was applied to reduce physiological and motion-related noise (Whitfield-Gabrieli and Nieto-Castanon, 2012) and to improve interpretability of the resulting correlation patterns (Chai et al., 2012, Saad et al., 2013). Following linear detrending, BOLD time series data were band-pass filtered between 0.008 and 0.09 Hz (Fox et al., 2005).

### 2.5 Functional connectivity analyses

Voxel-to-voxel covariance matrices for each subject were calculated in the first-level analysis, which further served to compute ICC (Martuzzi et al., 2011) and ILC (Deshpande et al., 2009) indices. ICC reflects the squared sum of mean connections of a given voxel with all the remaining voxels in the brain. ILC reflects the average correlation between a given voxel and its neighboring voxels. Compared to other local coherence approaches, ILC is independent of image resolution and a predefinition of the neighborhood is unnecessary. ILC also seems to be tissue-specific and largely independent of physiological noise. An 8-mm convolution kernel was applied to determine ILC bounds. In the second-level analysis, random-effects analysis of covariance was employed to conduct between-group comparisons. Correlation coefficients were *z*-transformed. First, case–control group comparisons were conducted using a two-sample *t*-test in CONN for both ICC and ILC measures. Second, linear regression analyses were conducted in CONN to test for associations of ICC and ILC with RA, PA, and CU scores separately. All regression analyses were performed at the whole-brain level and within the cases only. All analyses included study sites (8 dummy-coded variables), parent-reported ADHD symptoms (Broulidakis et al., 2016), and self-reported anxiety levels (Gomez et al., 2014, Leung et al., 2006, Motzkin et al., 2011) as covariates of no interest, given their crucial role in behavioral studies on aggression (Dadds et al., 2018, Fanti et al., 2013, Frick, 2016, Guelker et al., 2014). Additional sensitivity analyses including further covariates are provided in Supplementary Material S4. For all results, an uncorrected height threshold of *p* < 0.001 combined with a false-discovery-rate (FDR)-corrected cluster threshold of *p* < 0.05 was applied.

### 2.6 Exploratory analyses

In addition to the primary analyses regressing the RA and PA scores, we also explored the impact of general aggression (RPQ total score) as well as the effects of RA and PA while simultaneously controlling for the variance in PA and RA, respectively (as in Naaijen et al., 2020 for structural MRI analyses of the current study). This type of analysis relates to potential suppressor effects of other variables described previously in the literature on child/adolescent aggression (Ibrahim et al., 2021, Ibrahim et al., 2019, Lozier et al., 2014, Sebastian et al., 2014).

To address the heterogeneity of CU traits, we conducted exploratory analyses for CU subdimensions (callousness, uncaring, and unemotional). Finally, we performed additional analyses to examine the main effects of RA, PA, and general aggression on rsFC while controlling for CU traits.

## 3 Results

### 3.1 Behavioral results

Sample characteristics are summarized in detail in Table 1 (see also Werhahn et al., 2021 and Supplementary Material Table S3). Forty-eight cases had a diagnosis of ODD, twenty-five cases of CD and ODD, and seven cases of CD. Seventy-seven cases presented with scores in the clinical range (*T* > 70) on the aggression or rule-breaking behavior subscale of the CBCL, and forty-one cases scored in the clinical range on both subscales. Thirty-eight cases had an aggression score in the clinical range but no DSM diagnosis. These cases showed comparable RA values (M = 12.67, SD = 5.62) and CU traits (M = 33.61, SD = 10.01) and lower PA scores (M = 3.91, SD = 4.00) compared to cases with a DSM diagnosis (RA, M = 12.50, SD = 4.87; PA, M = 5.27, SD = 5.37; CU traits, M = 33.71, SD = 10.25). Most participants presented with comorbid ADHD symptoms (*n* = 103), while 66 cases had anxiety problems. Seventy cases were receiving medication. Aggression dimensions (RA, PA, CU traits) correlated with comorbidities of interest (ADHD inattention and hyperactivity/impulsivity, anxiety) in cases only for RA with anxiety symptoms (*r* = 0.32, *p* = 0.004), and for CU traits with ADHD inattention (*r* = 0.32, *p* = 0.001). Further associations among sample characteristics are reported in Supplementary Material Table S4.

**Table 1:** Sample characteristics.

	Cases (*n* = 118)	Controls (*n* = 89)
Age (Mean ± SD)	13.23±2.68	13.40±2.49
Sex, m/f (*N*) ^a^	99/19	51/38
IQ (Mean ± SD) ^a^	100.78±11.00	106.64±10.42
Handedness, left/right (*N*)	16/95	10/77
CD plus ODD Diagnosis (*N*)	25	
ODD Diagnosis (*N*)	48	
CD Diagnosis (*N*)	7	
ADHD Diagnosis (*N*)	29	
CBCL – Aggression *T*-score (Mean ± SD)	74.46±10.10	
CBCL – Rule-breaking *T*-score (Mean ± SD)	69.00±12.14	
SNAP-IV – Inattention (Mean ± SD) ^b^	15.47±6.06	2.00±4.11
SNAP-IV – Hyperactivity/Impulsivity (Mean ± SD) ^b^	12.00±5.95	1.00±3.17
YSR – Anxiety Problems (Mean ± SD) ^a^	3.00±2.26	2.00±2.04
ICU – Total Score (Mean ± SD) ^b^	33.68±10.16	21.00±8.70
RPQ – Reactive Aggression (Mean ± SD) ^b^	12.55±5.09	5.00±3.48
RPQ – Proactive Aggression (Mean ± SD) ^b^	3.00±5.01	0.82±1.45
Medication use (*N*) Stimulants Neuroleptics- Antidepressants	70 52 182	- - --

Values are means with standard deviations or counts. Abbreviations: IQ, intelligence quotient; CD, conduct disorder; ODD, oppositional defiant disorder; ADHD, attention-deficit hyperactivity disorder; CBCL, Child Behavior Checklist; SNAP-IV, Swanson, Nolan and Pelham Teacher and Parent Rating Scale; YSR, Youth Self Report; ICU, Inventory of Callous-Unemotional Traits, parent report; RPQ, Reactive and Proactive Aggression Questionnaire; RMS-FD, root mean square framewise displacement. Diagnoses are derived from the Kiddie-Schedule for Affective Disorders and Schizophrenia, present and lifetime version. Medication use was assessed from parental reports. Significant differences between groups are indicated by lowercase letters. ^a^*p* < 0.01, ^b^*p* < 0.001.

### 3.2 Voxel-to-voxel functional connectivity

Analyses of group differences yielded significant results only after exclusion of ADHD symptoms as a covariate (i.e. controlling only for site and anxiety symptoms; Supplementary Table S5). Cases showed lower ILC in a cluster including the bilateral frontal pole extending to the medial frontal cortex (t(142) = -6.12, p-FDR < 0.05; x  = 4, y = 66, z = -18). Moreover, cases demonstrated higher ICC in a cluster including the right occipital pole (t(142) = 5.19, p-FDR < 0.05; x  = 26, y = -102, z = 2). The ILC effect survived additional controlling for age, sex, IQ, and medication, but not handedness (t(131) = -5.67, p-FDR < 0.05; x  = -10, y = 56, z = –22). The ICC effect survived additionally controlling for age and sex (t(140) = 5.35, p-FDR < 0.05; x  = 26, y = -102, z = 2).

**Fig. 1:**
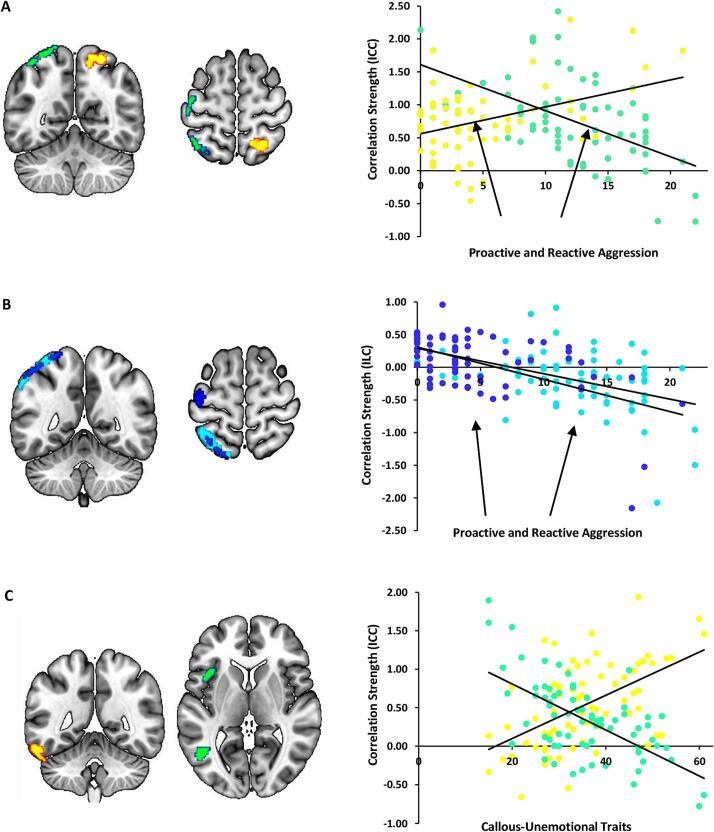
Whole-brain voxel-to-voxel connectivity correlates of reactive (RA) and proactive (PA) aggression as well as callous-unemotional (CU) traits. **A.** Higher RA and PA scores were associated with decreased versus increased intrinsic correlation contrast (ICC) in the parietal lobe and occipital cortex, colored in green and yellow, respectively. Moreover, a cluster in the left central gyrus exhibited RA-specific decreased ICC. **B.** Higher RA and PA scores were associated with decreased integrated local correlation (ILC) in regions colored in light and dark blue, respectively. Additionally, a region in the left central gyrus exhibited a PA-specific decrease in ILC. **C.** Higher CU traits were positively associated with ICC in the left inferior temporal gyrus (yellow), while a negative association was evident for the medial and inferior temporal gyrus and frontal and central opercular regions in the left hemisphere (green). ICC and ILC values (*y*-axis) are plotted against scores of RA and PA and CU traits (*x*-axis). The *z*-values are Fisher-transformed correlation coefficients, averaged across all observed voxels in cases comprising more than one cluster. The statistical threshold is *p* < 0.001, with a false discovery rate (FDR) cluster-level correction (*p* < 0.05). (For interpretation of the references to colour in this figure legend, the reader is referred to the web version of this article.)

The analysis with aggression scores showed that with higher levels of RA, ICC was decreased in the left superior parietal lobe and lateral occipital cortex in the left hemisphere, while, with higher levels of PA, ICC increased in these regions in the right hemisphere. The RA-related cluster extended to the left central gyrus, while the PA-related cluster extended to the precuneus (all p-FDR < 0.05; Fig. 1A, Supplementary Table S6). Moreover, with increasing levels of both RA and PA symptoms, ILC was decreased in left hemispheric clusters, including superior portions of the parietal lobe and lateral occipital cortex along with the supramarginal gyrus, extending to the angular gyrus. The PA-related cluster extended to the left central gyrus (all p-FDR < 0.05; Fig. 1B, Supplementary Table S7). Furthermore, with higher levels of CU traits, ICC was increased in a left hemispheric cluster including the inferior temporal gyrus and the cerebellum. In contrast, ICC was decreased in left hemispheric clusters that extended from the inferior temporal gyrus and middle temporal gyrus and inferior lateral occipital cortex to frontal and central opercular areas, including the insula lobe (all p-FDR < 0.05; Fig. 1C, see Supplementary Table S6).

### 3.3 Exploratory analyses

We identified a negative association between general aggression and ILC in the superior parietal lobule, supramarginal gyrus, pre-central/post-central gyrus, and superior frontal gyrus (Supplementary Table S7). No significant association was identified between general aggression and ICC.

We additionally examined the suppressor effect of aggression dimensions on ICC and ILC. Our results revealed a significant negative association between RA and ICC in the right pre-central/post-central gyrus when controlling for the effect of PA (Supplementary Fig. S2), and a significant positive association between PA and ICC in the left lateral frontal cortex and supplementary motor area when controlling for the effect of RA (Supplementary Fig. S2). For ILC analyses, the previously identified RA and PA main effects became statistically non-significant when controlled for PA and RA, respectively (Supplementary Fig. S3). We found similar results for the main effect of RA and PA when controlling for CU traits (Supplementary Tables S8 and S9, Supplementary Figs. S4 and S5).

Exploratory analyses for CU subdimensions are provided in Supplementary Tables S6 and S7. Among these three subdimensions, only the relationship with the uncaring facet showed significant effects. Positive associations were found in the pre-/post-central gyrus for ICC, and a negative association was found in the cerebellum for ILC. These effects did not overlap with those observed for the CU total score.

## 4 Discussion

The present study advances previous research on the neural basis of aggression using whole brain rsFC analyses without any particular a priori regions in a large sample of children and adolescents. Comorbid anxiety and ADHD symptoms were taken into account as possible modulators of aggressive dimensions. Between-group differences were detectable only after removing the control for ADHD symptoms, resulting in voxel-wise rsFC (ICC/ILC) findings in frontal clusters, which are typically implicated in cognitive control. Both RA and PA were correlated with voxel-wise rsFC alterations in superior parts of the parietal lobe, which are linked to attentional control. Our analyses also yielded disparate patterns for RA and PA in the left central gyrus and/or precuneus, which are implicated in aggression and cognitive control. Distinct CU trait-specific ICC associations were observed in temporal and cerebellar regions, which are known to mediate fear, morality, and reward processing.

Importantly, aggression subtype-specific rsFC findings were evident after additionally controlling for both ADHD and anxiety symptoms in the dimensional analyses. This suggests that these symptoms may have modulatory effects that can obscure aggression subtype-specific patterns. This further underlines the importance of considering both ADHD and anxiety in research on aggression. The crucial role of ADHD (Broulidakis et al., 2016, Uytun et al., 2017) and anxiety (Motzkin et al., 2011) in subtyping aggression is also substantiated by previous psychophysiological experiments (Fanti, 2018). Additionally, studies focused on aggressive behavior showed a correlation between anxiety symptoms and RA and PA (Fite et al., 2014, Fung et al., 2015) or RA symptoms alone (Marsee et al., 2008, Vitaro et al., 2002), as in our sample. In other studies, RA has been linked to anxiety and ADHD problems(Smeets et al., 2017) and internalizing symptoms (Fite et al., 2014). Multiple therapeutic components may be incorporated to relieve RA symptoms, including addressing anxiety, emotional problems, and hostile attributional biases, as RA symptoms may be manifestation of threat-circuit hypersensitivity (Blair et al., 2014). On the other hand, CU traits seem typically associated with reduced anxiety (Eisenbarth et al., 2016, Frick et al., 2014). Individuals with CU traits and co-occurring high levels of anxiety might thus represent a distinct phenotype with different clinical outcomes (Fanti et al., 2013) and neural correlates (Sethi et al., 2018). Moreover, within cases, we found generally low correlations of RA and PA levels and CU traits with ADHD and anxiety levels, which might further indicate heterogeneous underlying profiles associated with these forms of aggression. Overall, our results emphasize the value of differentiating between aggression subtypes and considering comorbid anxiety and ADHD symptoms. A similar conclusion may be reached from the case–control analysis. In our group analysis, cases exhibited rsFC differences in the medial frontal cortex compared to controls, conforming with our previous seed-based rsFC results (Werhahn et al., 2021), but only without controlling for ADHD symptoms. This suggests that ADHD symptoms affected prefrontal connectivity, consistent with the important role of the frontal lobe in ADHD and cognitive control (Ridderinkhof et al., 2004). The importance of considering anxiety levels in this context is in line with task-based fMRI studies in antisocial youths (Byrd et al., 2014), although the modulatory role of ADHD was not confirmed in previous studies (Broulidakis et al., 2016, Werhahn et al., 2021).

Our results suggest overlapping rsFC alteration patterns of RA and PA in brain regions involved in attention, decision-making, emotions, and CU-related traits. Thus, RA- and PA-related ICC was decreased in clusters including superior parts of the parietal lobe and lateral occipital cortex, however, in different hemispheres. These brain areas have been implicated in previous aggression-related rsFC studies. Decreased ILC was observed in the angular gyrus, where altered rsFC was reported also with antisocial personality disorder (Tang et al., 2016), and CU-related traits (Espinoza et al., 2018). Activity of the angular gyrus as part of the DMN has been associated with CU-related traits during emotional processing (Anderson et al., 2017) and with moral behaviors (Boccia et al., 2017, Fumagalli and Priori, 2012, Pujol et al., 2012). The lateral occipital cortex has been implicated in reduced functional activity during rest in adolescents with CD. The superior parietal lobe has been linked to attentional control (Bisley and Goldberg, 2010), learning from reward and punishment (Finger et al., 2011), and fearful expressions (Marsh et al., 2008, Peraza et al., 2015).

In line with behavioral studies (Fite et al., 2010, Marsee et al., 2011, Marsee and Frick, 2007) and our previous imaging study (Werhahn et al., 2021), we found distinct rsFC patterns associated with RA and PA symptoms. They involved the precuneus and central gyrus, which were previously linked to different forms of aggressive behavior and cognitive control-related processes known to be impaired in disruptive behavior (Blair et al., 2018). For example, adolescents with CD showed decreased postcentral gyrus rsFC (Cao et al., 2018, Lu et al., 2021a, Lu et al., 2021b, Lu et al., 2015). The precuneus, as part of the DMN, has been linked to self-reflection (Cavanna, 2007) and moral reasoning (Boccia et al., 2017), but also cognitive control and aggressive interactions (Fanning et al., 2017). In contrast with the neural findings delineated in this study and the correlation between CU traits and both RA and PA symptoms (Feilhauer et al., 2012, Pechorro et al., 2017), RA- and PA-related rsFC did not overlap with CU trait-related patterns. The strength of connectivity between a seed in the salience network projecting to the precuneus and central gyrus was previously found to be associated with CU sub-dimensions (Werhahn et al., 2021). Moreover, aberrant rsFC of the precuneus has been linked to impulsivity (Lu et al., 2017a), ADHD, and severe temper outbursts (Roy et al., 2018), but also to CU-related traits (Cao et al., 2019). During rest, the precuneus has previously been observed to show altered functional connectivity in male adolescents with CD (Zhou et al., 2016) and in male juvenile offenders (Chen et al., 2015) as well as in antisocial (Tang et al., 2016, 2013) and psychopathic adults (Motzkin et al., 2011, Philippi et al., 2015). Differences in rsFC of the precentral gyrus have also been reported in psychopathic adults (Espinoza et al., 2018, Tang et al., 2016).

In this study, CU traits were associated with connectivity measures in the left hemispheric inferior temporal gyrus, middle temporal gyrus, opercular regions, and the cerebellum. These findings align with previous studies using other connectivity methods, in which male adolescents also exhibited CU-related differences in the inferior temporal gyrus and middle temporal gyrus (Aghajani et al., 2016, Thijssen and Kiehl, 2017) as well as CD-related diminished activity in the right middle temporal gyrus (Wu et al., 2017), suggesting altered neural synchronization in these regions. The role of the middle temporal gyrus in this context has been further substantiated by the fact that this region exhibited the highest discriminating power among the DMN nodes to distinguish individuals with antisocial personality disorder from controls, while analysis of the cerebellum enabled the best differentiation overall (Tang et al., 2013). Additionally, other studies reported decreased regional homogeneity (a measure of local brain coherence) in the middle temporal gyrus of adults with antisocial personality disorder (Tang et al., 2013) and CU-specific rsFC decreases in prison inmates (Espinoza et al., 2018). Cerebellar and opercular regions have been implicated in rsFC alterations in adults with psychopathy (Philippi et al., 2015), and juvenile offenders (Aghajani et al., 2016). There is also evidence for the engagement of the left middle temporal gyrus in the theory of mind (Bzdok et al., 2012) and the CU-level-dependent fear response (Sebastian et al., 2014). The inferior temporal gyrus and middle temporal gyrus are also implicated in moral reasoning (Boccia et al., 2017), emotions (Alegria et al., 2016, Anderson et al., 2017), and reward processes (Crowley et al., 2010) along with the bilateral cerebellum (Veroude et al., 2016). Furthermore, exploratory analyses on the CU subdimensions only revealed an association between the uncaring facet and rsFC pattern. This effect did not overlap with the effect of CU total scores, supporting dimensional heterogeneity of CU traits. However, our results are exploratory in nature, and future studies can examine differential rsFC patterns in relation to CU subgroups, similar to prior work focused on brain structure (Ibrahim et al., 2021). This approach may help in identifying subtype-specific associations in rsFC.

Our multi-center study included a large sample of both male and female child and adolescent cases and controls. The study was not restricted to the inclusion of conduct disorder, given that CU traits are present in only a minority of children with CD (Frick, 2016). Moreover, we focused on RA and PA aggression symptoms and CU traits, applied unrestricted whole-brain analysis of rsFC, and controlled for ADHD and anxiety symptoms. Our results might enable conclusions about aggression subtypes of aggressive youths, with potential implications for clinical practice. The study also has limitations. Some results may have been influenced by data heterogeneity owing to data collection at different sites. Yet, the large sample size and the multi-center design increases the generalizability of our findings, and study site was accounted for as a covariate in each analysis. However, including site as a covariate of no interest in the analysis may have caused some loss of power. Furthermore, the 38 cases without a DSM diagnosis exhibited lower PA scores, however, the sub-analysis yielded comparable aggression subtype-specific associations. Lastly, although we included participants from both sexes, our sample predominantly consisted of males. This might restrict the generalizability of our results, although we found similar effects when controlled for sex (see Supplementary Material).

## 5 Conclusions

The current study showed both distinct and partially overlapping brain connectivity patterns of aggression subtypes and their modifying factors. Considering comorbid symptoms seems crucial for this process. The identified brain regions have been implicated in emotion regulation and moral-related behaviors. As a future research endeavor, investigating task-based alterations for the aggression subtypes could enhance their biological understanding. A transfer of aggression subtype-specific knowledge to clinical practice should be encouraged by tailoring existing therapeutic approaches (Umbach et al., 2015, Wilkinson et al., 2016). In these applications, neurobiological interventions, such as real-time fMRI/arousal-biofeedback training (Aggensteiner et al., 2023) or transcranial direct current stimulation (Chan et al., 2022), may become promising treatment strategies.

## Declaration of Competing Interest

The authors declare that they have no known competing financial interests or personal relationships that could have appeared to influence the work reported in this paper.

## Data Availability

Data will be made available on request.
