# Supplementary material for: Different whole-brain functional connectivity correlates of reactive-proactive aggression and callous-unemotional traits in children and adolescents with disruptive behaviors
Source: Neuroimage Clin. 2023 Nov 13;40:103542. doi: 10.1016/j.nicl.2023.103542 (PMC10701077; doi:10.1016/j.nicl.2023.103542)
Supplement: Supplementary data 1 [file mmc1.docx]

**Supplementary Material**

Different Whole-Brain Functional Connectivity Correlates of Reactive-Proactive Aggression and Callous-Unemotional Traits in Children and Adolescents with Disruptive Behaviors

**S1. Participants**

One hundred eighteen children and adolescents with a DSM diagnosis of conduct disorder (CD) and/or oppositional defiant disorder (ODD) and/or aggression scores in the clinical range as well as eighty-nine age- and handedness-matched healthy controls were included in the present study. Recruitment occurred across nine different sites in Europe at resident hospitals, ambulatories, and eligible (boarding) schools. The participating centers were as follows: Radboud University Medical Center and the Donders Center for Cognitive Neuroimaging, Nijmegen, The Netherlands; Department of Child and Adolescent Psychiatry, University Medical Centre Groningen, The Netherlands; Central Institute of Mental Health, Medical Faculty, Mannheim/Heidelberg University, Mannheim, Germany; University of Ulm, Department of Child and Adolescent Psychiatry/Psychotherapy and Department of Psychiatry III, Ulm, Germany; Department of Child Psychiatry, and the Centre for Neuroimaging Sciences, Institute of Psychiatry, Psychology and Neuroscience, King`s College London, London, England; Institut d'Investigacions Biomèdiques August Pi i Sunyer and Hospital Clinic de Barcelona, Barcelona, Spain; Instituto de Investigación Sanitaria Gregorio Marañón, Child and Adolescent Psychiatry Department of Gregorio Marañòn General University Hospital, Madrid, Spain; Department of Child and Adolescent Psychiatry and Psychotherapy, University Zurich and MR Center, Psychiatric University Hospital, Zurich, Switzerland; IRCCS Santa Lucia Foundation, Rome, Italy.

Besides a DSM diagnosis of CD, ODD, and/or an aggression or rule-breaking behavior subscale score in a clinical range (*T* > 70) according to the Child Behavior Checklist (CBCL), Youth Self Report (YSR), or Teacher Report Form (TRF), a further inclusion criterion for the case group was a lack of medication or a stable medication for at least two months. Exclusion criteria for cases was a primary DSM diagnosis of depression, anxiety, psychosis, or bipolar disorder and for the typically developed comparison group a DSM diagnosis or clinically relevant scores on the CBCL, YSR, or TRF. Further exclusion criteria for all participants were an IQ score <80 as measured by the Wechsler Intelligence Scale for Children, Fourth Edition (WISC-IV) and common contraindications for MRI scanning, such as braces or metal implants. Additionally, an anxiety score >8 on a Visual Analogue Scale ranging from 1 to 10 before scanning led to exclusion, as anxiety caused by the scanner could impair data quality and alter neural activity (Muehlhan et al., 2011). Participants had sufficient native language skills according to the assessing country. All sites obtained ethical approval separately. After eligible individuals received information about the study procedure, participants and their parents or legal representatives gave written informed consent.

**S2. Assessment Tools and Study Procedure**

Diagnostic assessments and magnetic resonance (MR) measurements took place on different dates to minimize the burden on the participants. During the first appointment, parents/primary caregivers and children/youths were assessed by trained (clinical) psychologists or supervised interns separately with the semi-structured interview Kiddie-Schedule for Affective Disorders and Schizophrenia, present and lifetime version K-SADS (Kaufman et al., 1997). The full supplementary module of the specific disorder supported by the assessment followed positively answered questions. Diagnoses resulted from clinical evaluation after self- and parent-reports. Parents/primary caregivers and children/youths answered a questionnaire battery that included the following measures:

• CBCL: a parent-report questionnaire rating the child or adolescent on various behavioral and emotional problems (Achenbach, 1991).

• The Swanson, Nolan, and Pelham teacher and parent rating scale (SNAP-IV): a measure containing 26 items to assess attention deficit disorder (ADHD) and ODD symptoms from childhood to young adulthood (Swanson, 1992).

• The Inventory of Callous-Unemotional traits (ICU): a 24-item questionnaire ranging from 0 (not at all true) to 3 (definitely true) (Frick, 2004).

• The Reactive-Proactive Aggression Questionnaire (RPQ): a 23-item self-report measure of the frequency (never, 0; sometimes, 1; often, 2) of reactive (12 items) and proactive aggression (11 items) (Raine et al., 2006).

IQ was estimated according to four sub-tests derived from the Wechsler Intelligence Scale for Children (WISC-IV) (Wechsler, 2003): block design, similarities, vocabulary, picture completion. Additionally, the digit span subtest was assessed.

The test battery implemented in the framework of the Aggressotype/MATRICS projects included additional questionnaires, which are not relevant for this work. These instruments were as follows: Modified Aggression Scale (Kay et al., 1988), Longterm difficulties self- and parent-report (Ormel et al., 2012), Pubertal Developmental Scale (Petersen et al., 1988), Antisocial Behavior Scale (Slot et al., 2000), Strengths and Difficulties Questionnaire (Goodman, 1997), YSR and TRF as further parts of Achenbach System of Empirically Based Assessment (Achenbach, 1991), as ICU (Frick, 2004) self- and parent-reports, and cognitive tests (Probabilistic Reversal Learning Task (Cools et al., 2002) and three tasks from the Cambridge Neuropsychological Test Automated Battery (Robbins et al., 1994): Emotion Recognition Task, Delayed Matching to Sample and Rapid Visual Information Sampling). Blood- or saliva-samples were collected for further analyses. Two sites additionally conducted a short psychophysiological measurement (including measures of heart rate (pulse), skin conductance level, and EEG before, during, and after an emotional paradigm).

All children/youths were prepared for MR scanning with their parents/primary caregivers. They were presented with MR sounds and visited a dummy scanner and/or watched a MR information video to become familiar with the scanner environment. If the participants or parent/primary caregiver reported an anxiety rating of >8 on a Visual Analogue Scale (VAS) ranging from 1 (no anxiety) to 10 (very high anxiety) related to entering the MR scanner, this led to an exclusion from further investigation.

Participants completed a MR safety form and a short pre-scanning questionnaire (regarding tobacco and cannabis consumption, genetic diseases in the family, etc.) and then practiced the following fMRI tasks on a laptop: passive avoidance task (Finger et al., 2011), emotional matching task (Via et al., 2014), and stop signal task (Rubia et al., 2011). Participants gave a saliva sample right before MRI scanning. Female pregnancy was ruled out by a urine pregnancy test or based on self-reports. The first part of MRI scanning session included a T1-weighted anatomical image, the three fMRI tasks, and the fMRI resting state sequence. For the resting state scan, participants were instructed to look at a white crosshair against a black background and let their mind wander. After a break that included the collection of another saliva sample and a short questionnaire regarding the performance in the fMRI tasks, a T1-weighted anatomical scan was followed by two sequences of magnetic resonance spectroscopy and diffusion tensor imaging. After all study-related procedures, the monetary reward and travel costs were reimbursed. Additionally, participants received a picture of their anatomical MR scan. The current study reports resting state fMRI and psychometric data.

**S3. Image Acquisition**

Supplementary Tables 1 and 2 show the site-specific scanner information.

| **Supplementary Table 1.**  Structural MRI scan parameters across sites. | | | | | | | |
| --- | --- | --- | --- | --- | --- | --- | --- |
| **Scanner** | **Site** | **TR/TE/T1 (ms)** | **Flip angle** | **Field of view** | **Matrix RL/AP/slices** | **Voxel size (mm)** | **Acceleration factor** |
| Siemens | Nijmegen | 2300/2.98/900 | 9 | 256 | 212/256/176 | 1.0 × 1.0 × 1.2 | 2 |
|  | Mannheim | 2300/2.96/900 | 9 | 256 | 212/256/176 | 1.0 × 1.0 × 1.2 | 2 |
|  | Ulm | 2300/2.96/900 | 9 | 256 | 212/256/176 | 1.0 × 1.0 × 1.2 | 2 |
|  | Barcelona | 2300/2.98/900 | 9 | 256 | 212/256/176 | 1.0 × 1.0 × 1.2 | 2 |
|  | Madrid | 2300/2.98/900 | 9 | 256 | 212/256/176 | 1.0 × 1.0 × 1.2 | 2 |
|  | Rome | 2080/2.86/900 | 9 | 256 | 212/256/176 | 1.0 × 1.0 × 1.2 | 2 |
| Philips | Groningen | 2450/3.11/900 | 8 | 270 | 256/232/170 | 1.0 × 1.0 × 1.0 | 1.8 |
|  | Zurich | 2300/3.11/900 | 9 | 270 | 256/232/170 | 1.0 × 1.0 × 1.0 | 1.8 |
| GE | London | 2300/3.02/400 | 11 | 270 | 256/256/196 | 1.0 × 1.0 × 1.2 | 1.75 |

| **Supplementary Table 2.**  Resting state functional MRI scan parameters across sites. | | | | | | |
| --- | --- | --- | --- | --- | --- | --- |
| **Scanner** | **Site** | **TR, TE1/TE2/TE3 (ms)** | **Number of slices** | **Slice scan order** | **Voxel size (mm)** | **Duration (min)** |
| Siemens | Nijmegen | 2300, 12/28.4/44.8 | 33 | descending | 3.8 × 3.8 × 3.8 | 8:24 |
|  | Mannheim | 2300, 12/29/46 | 33 | descending | 3.8 × 3.8 × 3.8 | 8:24 |
|  | Ulm | 2300, 31 | 33 | descending | 3.8 × 3.8 × 3.8 | 8:23 |
|  | Barcelona | 2300, 12 | 33 | descending | 3.8 × 3.8 × 3.8 | 8:21 |
|  | Madrid | 2300, 13 | 36 | descending | 3.8 × 3.8 × 3.8 | 8:24 |
|  | Rome | 2080, 30 | 32 | ascending | 3.0 × 3.0 × 2.5 | 7:38 |
| Philips | Groningen | 2450, 8.01/22.02/36.02 | 45 | descending | 3.5 × 3.5 × 3.5 | 10:08 |
|  | Zurich | 2300, 13/31/49 | 33 | descending | 3.75 × 3.75 × 3.79 | 7:51 |
| GE | London | 2300, 11.8/31/48 | 33 | descending, interleaved | 3.45 × 3.45 × 4.20 | 8:15 |
|  |  |  |  |  |  |  |

**S4. Supplementary Results**

The distribution of demographic characteristics, diagnoses, and clinical aggression scores on behavioral measures across sites are shown in Supplementary Table 3. The investigated behavioral measures exhibited considerable standard deviations, which enabled the planned dimensional approach of aggression dimension-specific rsFC analyses.

In the whole sample, reactive aggression (RA) and proactive aggression (PA) symptoms were correlated positively with ADHD inattention subscale (*r* = 0.50 and *r* = 0.34, *p* < 0.001), ADHD hyperactivity/impulsivity subscale (*r* = 0.50 and *r* = 0.43, *p* < 0.001), and anxiety problems (*r* = 0.36, *p* < 0.001 and *r* = 0.19, *p* = 0.02). CU traits were also correlated positively with ADHD inattention and hyperactivity/impulsivity subscales (*r* = 0.59 and *r* = 0.49, *p* < 0.001) and with anxiety levels, but not reaching the significance threshold (*r* = 0.08, *p* > 0.05). Within cases, RA and PA symptoms were neither significantly correlated with ADHD inattention subscale (*r* = -0.01, *p* > 0.05 and *r* = -0.04, *p* > 0.05) nor hyperactivity/impulsivity subscale (*r* = 0.01, *p* > 0.05 and *r* = 0.12, *p* > 0.05). Only the positive correlation of RA (*r* = 0.32, *p* = 0.004), but not PA (*r* = 0.10, *p* > 0.05), with anxiety symptoms were statistically significant. CU traits were positively associated with ADHD inattention subscale (*r* = 0.32, *p* = 0.001) but with neither hyperactivity/impulsivity subscale (*r* = 0.13, *p* > 0.05) nor anxiety problems (*r* = -0.06, *p* > 0.05).

Cases showed positive correlations between RA and PA (*r* = 0.63, *p* < 0.001) and between CU traits and PA (*r* = 0.33, *p* = 0.001), while the positive association of CU traits and RA did not reach significance (*r* = 0.13, *p* > 0.05).

The average RMS-FD for cases was 0.12 mm (*SD* = 0.17 mm), while for controls it was 0.09 (*SD* = 0.18mm) and differed significantly (*p* < 0.01). In the whole sample, average RMS-FD was correlated positively with CU traits (*r* = 0.17, *p* < 0.05), while associations with CBCL rule-breaking and aggression subscales, reactive and proactive scores along with ADHD inattention and hyperactivity/impulsivity subscales, and anxiety problems did not reach statistical significance (*p* > 0.05) or even approaching significance. Within cases, there was no significant or trend level correlation between mean RMS-FD and these clinical characteristics. As cases and controls differed in average RMS-FD values, we conducted sensitivity analyses for our case-control differences in voxel-wise rsFC (where we additionally controlled for anxiety but not ADHD symptoms) and additionally included RMS-FD as a covariate. Compared to controls, cases showed comparably decreased ICC in a cluster including the right occipital pole (*t*(141) = -5.28, p-FDR < 0.05) and increased ILC in a left hemispheric frontal cluster including the bilateral frontal pole extending to the medial frontal cortex (*t*(141) = 6.23, p-FDR < 0.05).

For all voxel-to-voxel whole-brain rsFC analyses, a statistical threshold of *p* < 0.001 and a *p* < 0.05 cluster-level FDR correction for multiple comparisons was applied (see Supplementary Table 5 and 6). After primary analyses that included controlling for site, ADHD, and anxiety, we checked whether the observed results survived additional controlling for further covariates, including age, sex, IQ, medication, and handedness, subsequently, as they can possibly influence rsFC of resting state networks such as the default mode network (DMN) (Allen et al., 2011; Mak et al., 2017; Sherman et al., 2014) or functional activity (Liu et al., 2009) (see Supplementary Table 5 and 6).

Given the observed effects of the control for both ADHD and anxiety symptoms on aggression-related rsFC, we further explored their influence separately on rsFC within cases. Anxiety problems were associated negatively with intrinsic connectivity contrast (ICC) in a right hemispheric cluster including temporal pole and anterior divisions of the superior and middle temporal gyrus, when controlling for site and ADHD symptoms. Moreover, only the ADHD inattention but not hyperactivity subscale was positively linked to ICC in a cluster including the right superior lateral occipital cortex and also positively link to integrated local correlation (ILC) in the right hemispheric lingual gyrus, occipital fusiform gyrus, and cerebellum regions. Yet, these effects were only observed when controlling for site but not anxiety symptoms (all p-FDR < 0.05).

Furthermore, we explored rsFC patterns when controlling for neither ADHD nor anxiety symptoms. We found increased ILC in a cluster including the bilateral frontal pole only for cases compared to controls. Within cases, CU traits were associated with decreased ICC in a left hemispheric cluster including the middle temporal gyrus and inferior lateral occipital cortex (all p-FDR < 0.05).

After controlling for ADHD symptoms but not anxiety levels, only one cluster in the left central gyrus showing reduced ILC with higher proactive aggression ILC remained significant. Another cluster extending from the left hemispheric Heschl`s gyrus to the superior temporal gyrus exhibited increased ILC with higher levels of CU traits. When anxiety problems but not ADHD symptoms were controlled for, the majority of aggression subtype-specific rsFC patterns disappeared. Yet, the reactive aggression-specific decrease remained significant in one cluster including the left superior division of the parietal lobe. Furthermore, one left hemispheric cluster in the inferior temporal gyrus, middle temporal gyrus, and inferior lateral occipital cortex remained, showing decreased ICC with higher levels of CU traits (all p-FDR < 0.05).

Within cases, additional analyses showed negative associations between RA score and ICC in two clusters, which were also present after exclusion of 38 cases without a DSM diagnosis of CD and/or ODD. The positive associations with PA and ICC in another cluster remained significant as well after excluding the above subsample. Furthermore, the negative association between RA score and ILC remained significant. Lastly, we found significant negative associations between PA score and ILC in three clusters that also remained significant after the exclusion of cases without a diagnosis.

| **Supplementary Table 3.**  Distribution of demographic characteristics, diagnoses, and aggression scores across sites. | | | | |
| --- | --- | --- | --- | --- |
|  |  | Cases (*N* = 118) | HC (*N* = 89) |  |
| Nijmegen (*N* = 40) | Age | 13.55 ± 2.59 | 12.64 ± 1.96 |  |
|  | IQ | 100.41 ± 11.83 | 107.86 ± 12.45 |  |
|  | Sex, m/f | 14/4 | 14/8 |  |
|  | Medication | 10 | 0 |  |
| Groningen  (*N* = 16) | Age | 14.77 ± 2.54 | 12.39 ± 2.32 |  |
|  | IQ | 101.40 ± 11.63 | 101.85 ± 2.58 |  |
|  | Sex, m/f | 8/2 | 2/4 |  |
|  | Medication | 6 | 0 |  |
| Mannheim  (*N* = 33) | Age | 12.78 ± 2.42 | 12.97 ± 3.06 |  |
|  | IQ | 102.16 ± 10.13 | 116.32 ± 7.73 |  |
|  | Sex, m/f | 19/3 | 9/2 |  |
|  | Medication | 14 | 0 |  |
| Ulm (*N* = 18) | Age | 10.55 ± 2.38 | 13.59 ± 3.26 |  |
|  | IQ | 103.62 ± 17.55 | 101.41 ± 7.97 |  |
|  | Sex, m/f | 5/0 | 4/9 |  |
|  | Medication | 5 | 0 |  |
| London (*N* = 26) | Age | 14.67 ± 2.25 | 13.71 ± 2.05 |  |
|  | IQ | 97.16 ± 10.33 | 111.85 ± 11.01 |  |
|  | Sex, m/f | 15/0 | 8/3 |  |
|  | Medication | 12 | 1 |  |
| Barcelona  (*N* = 23) | Age | 12.99 ± 2.82 | 14.94 ± 2.31 |  |
|  | IQ | 102.57 ± 12.10 | 105.70 ± 6.59 |  |
|  | Sex, m/f | 10/4 | 5/4 |  |
|  | Medication | 7 | 0 |  |
| Madrid (*N* = 20) | Age | 14.29 ± 2.22 | 14.68 ± 1.84 |  |
|  | IQ | 98.73 ± 9.45 | 105.74 ± 9.31 |  |
|  | Sex, m/f | 10/3 | 5/2 |  |
|  | Medication | 10 | 0 |  |
| Zürich  (*N* = 20) | Age | 10.53 ± 1.87 | 11.75 ± 1.49 |  |
|  | IQ | 102.61 ± 11.20 | 99.60 ± 7.87 |  |
|  | Sex, m/f | 10/3 | 3/4 |  |
|  | Medication | 2 | 0 |  |
| Rome  (*N* = 11) | Age | 14.37 ± 2.60 | 16.12 ± 1.81 |  |
|  | IQ | 95.84 ± 8.83 | 97.23 ± 2.78 |  |
|  | Sex, m/f | 8/8 | 1/2 |  |
|  | Medication | 4 | 0 |  |
| *Note:* Values are means ± SD, or counts. HC = healthy controls. ODD CD ODD + CD     (additional) aggression in a clinical range (T > 70 on aggression subscales in Child Behavior Checklist)  Nijmegen  Groningen Mannheim  Ulm  London  Barcelona Madrid  Zürich  Rom   | | | | |
|  | | | | |

| **Supplementary Table 4.**  Bivariate correlations of aggression-related scores within cases. | | | | | | | | | | |
| --- | --- | --- | --- | --- | --- | --- | --- | --- | --- | --- |
|  | rule-break-ing | aggres-sion | ODD | CD | inatten-tion | hyper-activity/ impul-sivity | anxiety | CU traits | reactive aggres-sion | pro-active aggres-sion |
| rule-break-ing |  |  |  |  |  |  |  |  |  |  |
| aggres-sion | *r* = 0.47, *p* < 0.001 |  |  |  |  |  |  |  |  |  |
| ODD | *r* = 0.16,  *p* > 0.05 | *r* = 0.20,  *p* < 0.05 |  |  |  |  |  |  |  |  |
| CD | *r* = 0.38, *p* < 0.001 | *r* = 0.21,  *p* < 0.05 | *r* = 0.28,  *p* < 0.01 |  |  |  |  |  |  |  |
| inatten-tion | *r* = 0.19, *p* > 0.05 | *r* = 0.22,  *p* < 0.05 | *r* = -0.01,  *p* < 0.001 | *r* = 0.14,  *p* > 0.05 |  |  |  |  |  |  |
| hyper-activi-ty/ impul-sivity | *r* = 0.20,  *p = 0.05* | *r* = 0.26,  *p* < 0.01 | *r* = 0.28,  *p* < 0.01 | *r* = 0.05,  *p* > 0.05 | *r* = 0.55,  *p* < 0.001 |  |  |  |  |  |
| anx-iety | *r* = -0.02, *p* > 0.05 | *r* = -0.09  *p* > 0.05 | *r* = -0.02,  *p* > 0.05 | *r* = 0.07,  *p* > 0.05 | *r* = -0.16,  *p* > 0.05 | *r* = -0.14,  *p* > 0.05 |  |  |  |  |
| CU traits | *r* = 0.16, *p* > 0.05 | *r* = 0.34,  *p* < 0.001 | *r* = 0.18,   *p > 0.05* | *r* = 0.26,  *p* < 0.01 | *r* = 0.32,  *p* = 0.01 | *r* = 0.13,  *p* > 0.05 | *r* = -0.06,  *p* > 0.05 |  |  |  |
| reac-tive  aggres-sion | *r* = 0.05, *p* > 0.05 | *r* = 0.16,  *p* > 0.05 | *r* = 0.07,  *p* > 0.05 | *r* = 0.13,  *p* > 0.05 | *r* = -0.01,  *p* > 0.05 | *r* = 0.01,  *p* > 0.05 | *r* = 0.32,  *p* < 0.01 | *r* = 0.13,  *p* > 0.05 |  |  |
| pro-active aggres-sion | *r* = 0.14, *p* > 0.05 | *r* = 0.22,  *p* < 0.05 | *r* = 0.22,  *p* < 0.05 | *r* = 0.29,  *p* < 0.01 | *r* = -0.04,  *p* > 0.05 | *r* = 0.12,  *p* > 0.05 | *r* = 0.10,  *p* > 0.05 | *r* = 0.33,  *p* = 0.001 | *r* = 0.63,  *p* < 0.001 |  |
| *Note:* Rule-breaking and aggression *T*-scores derived from the Child Behavior Checklist. ODD and CD scores are according to the Kiddie-Schedule for Affective Disorders and Schizophrenia, present and lifetime version. Callous-unemotional (CU) traits, total score, are derived from the parent-reported Inventory of Callous-Unemotional traits; reactive and proactive aggression scores are according to the self-reported Reactive-Proactive Aggression Questionnaire. Inattention, hyperactivity/impulsivity scores are according to the Swanson, Nolan, and Pelham teacher and parent rating scale (SNAP-IV); anxiety problems are derived from the Youth Self Report. | | | | | | | | | | |

| **SUPPLEMENTARY TABLE 5.**  Significant clusters derived from the integrated local correlation (ILC) and intrinsic connectivity contrast (ICC) results for the case-control analyses. | | | | | | | | |
| --- | --- | --- | --- | --- | --- | --- | --- | --- |
|  |  | |  |  |  | Peak voxel  MNI coordinates | | |
|  | Brain region | | Hemisphere | Cluster size | *β*-value | *X* | *Y* | *Z* |
| 1  2 3 *    * | **Integrated Local Correlation**  No significant effects  No significant effects  No significant effects  *Negative Association* Controlling for site and anxiety symptoms  - Frontal Pole, Frontal Medial Cortex |  | L/R | 310 | -0.41 | 04 | 66 | -18 |
|  | Controlling for site, anxiety symptoms, age, sex, IQ, medication - Frontal Pole, Frontal Medial Cortex |  | L/R | 170 | -0.58 | -10 | 56 | -22 |
| 1  2 3  *  * | **Intrinsic Connectivity Contrast**  No significant effects  No significant effects  No significant effects  *Positive Association* Controlling for site and anxiety symptoms  - Occipital Pole |  | R | 80 | 0.41 | 26 | -102 | 02 |
|  | Controlling for site, anxiety symptoms, age, sex - Occipital Pole |  | R | 75 | 0.43 | 26 | -102 | 02 |
| *Note:* Brain regions and cytoarchitectonic coordinates of peak voxels are labeled according to the Anatomy toolbox for SPM using Montreal Neurological Space (MNI) anatomical coordinates. Statistical thresholds for the reported results are *p* < 0.001, false discovery rate (FDR) cluster-level corrected (*p* < 0.05). The following corrections were applied: (1) for site, ADHD, anxiety scores; (2) for site, age, sex, medication, IQ, handedness; (3) for site, age, sex, medication, IQ and handedness along with ADHD and anxiety scores; * for other variable sets, as specified. | | | | | | | | |

**
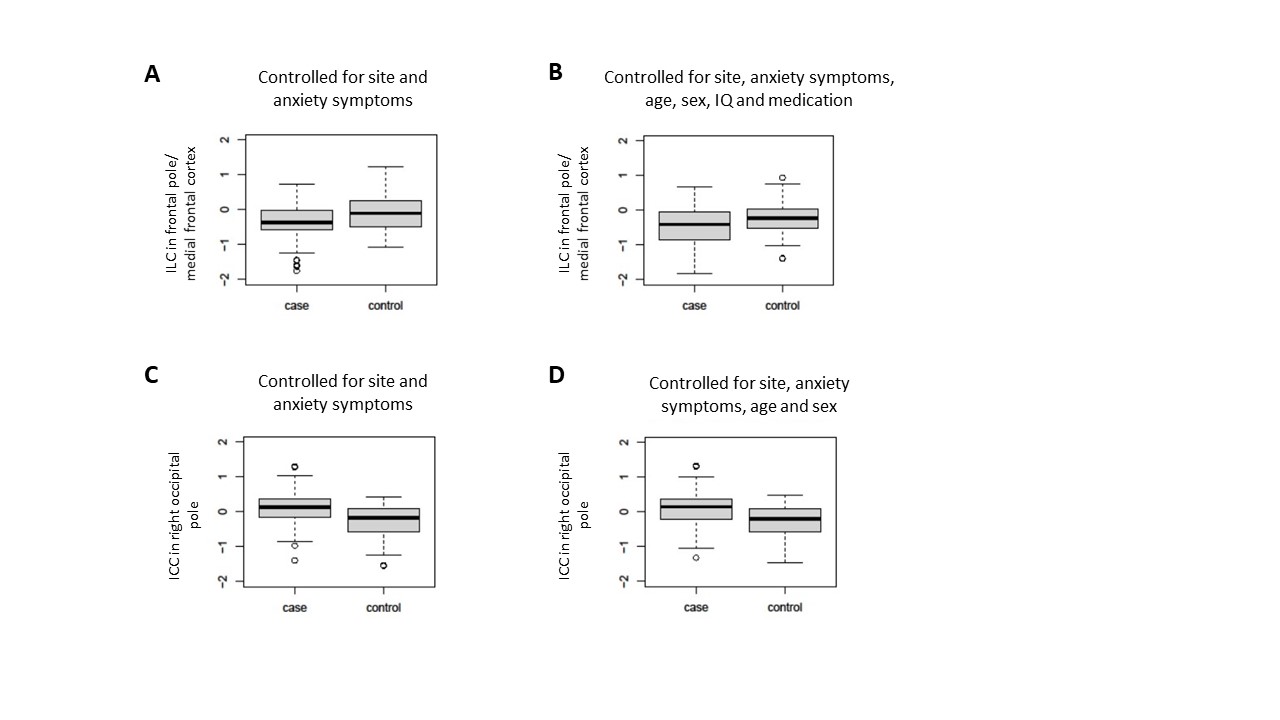
Supplementary Figure 1**. Boxplots of the ILC and ICC case-control differences. A. ILC values in the frontal pole/frontal medial cortex cluster (controlling for site and anxiety symptoms). B. ILC values in the frontal pole/frontal medial cortex cluster (controlling for site, anxiety symptoms, age, sex, IQ and medication). C. ICC values in the occipital pole cluster (controlling for site and anxiety symptoms). D. ICC values in the occipital pole cluster (controlling for site, anxiety symptoms, age and sex).

**SUPPLEMENTARY TABLE 6.**

Clusters and coordinates derived from intrinsic connectivity contrast (ICC) results related to reactive and proactive aggression, general aggression, and callous-unemotional (CU) traits.

|  |  |  |  |  |  | MNI Coordinates | | |
| --- | --- | --- | --- | --- | --- | --- | --- | --- |
|  | Behavioral  Measure | Brain region | Hemisphere | Cluster size | *β*-value | *X* | *Y* | *Z* |
| **1** | RPQ - RA | *Negative Association* - Superior Parietal Lobe - Postcentral Gyrus | L L | 117 61 | -0.48 -0.49 | -32 -48 | -56 -12 | 68 58 |
|  | RPQ- PA | *Positive Association* - Superior Parietal Lobe | R | 88 | 0.27 | 26 | -56 | 62 |
|  | RPQ- total | No significant results |  |  |  |  |  |  |
|  | ICU- total | *Positive Association* - Inferior Temporal Gyrus  *Negative Association* - Inferior Temporal Gyrus  - Insula Lobe | L  L L | 130  139 59 | 0.38  -0.41 -0.36 | -54  -48 -40 | -52  -60 16 | -20  00 08 |
|  | ICU- Callousness | No significant results |  |  |  |  |  |  |
|  | ICU- uncaring | *Positive Association* - Parahippocampal Gyrus, Temporal Fusiform Cortex  - Superior Frontal Gyrus | L  L | 106  63 | 0.56  0.47 | -26  -20 | -14  22 | -40  52 |
|  | ICU-unemotional | No significant results |  |  |  |  |  |  |
| **2** | RPQ – RA | No significant results |  |  |  |  |  |  |
|  | RPQ- PA | No significant results |  |  |  |  |  |  |
|  | RPQ- total | No significant results |  |  |  |  |  |  |
|  | ICU- total | *Negative Association*  - Middle Temporal Gyrus, Lateral Occipital Pole | L | 95 | -0.34 | -46 | -60 | 00 |
|  | ICU- Callousness | No significant results |  |  |  |  |  |  |
|  |  |  |  |  |  |  |  |  |
| **Supplementary Table 6 continued** | | | | | | | | |
|  | ICU- uncaring | *Positive Association*  - Pre-central/Post-central Gyrus  - Central Opercular Cortex | L  R | 123  72 | 0.43  0.43 | -38  68 | -18  -16 | 54  10 |
|  | ICU-unemotional | No significant results |  |  |  |  |  |  |
|  | ICU- uncaring | *Positive Association* - Parahippocampal Gyrus, Temporal Fusiform Cortex  - Superior Frontal Gyrus | L  L | 106  63 | 0.56  0.47 | -26  -20 | -14  22 | -40  52 |
|  |  |  |  |  |  |  |  |  |
|  | ICU-unemotional | No significant results |  |  |  |  |  |  |
| **3** | RPQ – RA | No significant results |  |  |  |  |  |  |
|  | RPQ- PA | *Positive Association* - Superior Parietal Lobe | R | 94 | 0.31 | 26 | -56 | 62 |
|  | RPQ- total | No significant results |  |  |  |  |  |  |
|  | ICU- total | *Negative Association* - Inferior Temporal Gyrus - Insula Lobe | L L | 132 67 | -0.41 -0.35 | -48 -40 | -60 16 | 00 08 |
|  | ICU- Callousness | No significant results |  |  |  |  |  |  |
|  | ICU- uncaring | *Positive Association* - Pre-central/Post-central Gyrus  *Negative Association* - not-labeled | L  L | 78  78 | 0.53  -0.62 | -38  -20 | -16  -56 | 54  44 |
|  |  |  |  |  |  |  |  |  |
|  | ICU-unemotional | No significant results |  |  |  |  |  |  |

*Note:* Brain regions and cytoarchitectonic coordinates of peak voxels for callous-unemotional (CU) traits and reactive and proactive aggression are labeled according to the Anatomy toolbox for SPM using Montreal Neurological Space (MNI) anatomical coordinates. Statistical thresholds for the reported results are *p* < 0.001, false discovery rate (FDR) cluster-level corrected (*p* < 0.05) for multiple comparisons. The β-values represent *z*-standardized correlation coefficients. The following corrections were applied: (1) for site, ADHD, anxiety scores; (2) for site, age, sex, medication, IQ, handedness; (3) for site, age, sex, medication, IQ and handedness along with ADHD and anxiety scores. Abbreviations: ICU, Inventory of Callous-Unemotional Traits; RA, reactive aggression; RPQ, Reactive-Proactive Aggression Scale; PA, proactive aggression.

**SUPPLEMENTARY TABLE 7.**

Clusters and coordinates derived from integrated local correlation (ILC) results related to reactive and proactive aggression, general aggression, and callous-unemotional (CU) traits.

|  |  |  |  |  |  | MNI Coordinates | | |
| --- | --- | --- | --- | --- | --- | --- | --- | --- |
|  | Behavioral  Measure | Brain region | Hemisphere | Cluster size | *β*-value | *X* | *Y* | *Z* |
| **1** | RPQ - RA | *Negative Association* - Superior Parietal Lobe | L | 288 | -0.33 | -32 | -54 | 68 |
|  | RPQ- PA | *Negative Association* - Precentral Gyrus - Superior Parietal Lobe - Inferior Parietal Lobe | L L L | 101 85 83 | -0.28 -0.21 -0.22 | -42 -26 -44 | -16 -56 -48 | 62 70 60 |
|  | RPQ- total | *Negative Association* - Superior Parietal Lobule, Supramarginal Gyrus - Pre-central/Post-central Gyrus | L  L | 362  119 | -0.28  -0.32 | -30  -42 | -56  -16 | 68  62 |
|  | ICU- total | No significant results |  |  |  |  |  |  |
|  | ICU- Callousness | No significant results |  |  |  |  |  |  |
|  | ICU- uncaring | No significant results |  |  |  |  |  |  |
|  | ICU-unemotional | No significant results |  |  |  |  |  |  |
| **2** | RPQ - RA | No significant results |  |  |  |  |  |  |
|  | RPQ- PA | No significant results |  |  |  |  |  |  |
|  | RPQ- total | No significant results |  |  |  |  |  |  |
|  | ICU- total | No significant results |  |  |  |  |  |  |
|  | ICU- Callousness | No significant results |  |  |  |  |  |  |
|  | ICU- uncaring | *Negative Association* - Cerebellum | L | 121 | -0.55 | -28 | -54 | -54 |
|  | ICU-unemotional | No significant results |  |  |  |  |  |  |
| **3** | RPQ - RA | *Negative Association* - Superior Parietal Lobe | L | 187 | -0.36 | -38 | -50 | 64 |
|  | | | | | | | | |
| **Supplementary Table 7 continued** | | | | | | | | |
|  | RPQ- PA | *Negative Association* - Inferior Parietal Lobe - Precentral Gyrus - Superior Frontal Gyrus - Precentral Gyrus | L L R L | 201 160 139 99 | -0.24 -0.30 -0.22 -0.26 | -44 -42 24 -60 | -48 -16 28 -06 | 60 62 60 36 |
|  | RPQ- total | *Negative Association* - Superior Parietal Lobule, Supramarginal Gyrus - Pre-central/Post-central Gyrus - Superior Frontal Gyrus | L  L  R | 312  182  133 | -0.30  -0.37  -0.27 | -44   -44  24 | -48  -16  28 | 60  60  60 |
|  | ICU- total | No significant results |  |  |  |  |  |  |
|  | ICU- Callousness | No significant results |  |  |  |  |  |  |
|  | ICU- uncaring | No significant results |  |  |  |  |  |  |
|  | ICU-unemotional | No significant results |  |  |  |  |  |  |

*Note:* Brain regions and cytoarchitectonic coordinates of the peak voxels are labeled based upon the Anatomy toolbox for SPM using Montreal Neurological Space (MNI) anatomical coordinates. The statistical threshold for the reported results is *p* < 0.001, false discovery rate (FDR) cluster-level corrected (*p* < 0.05) for multiple comparisons. Β-values represent *z*-standardized correlation coefficients. The following corrections were applied: (1) for site, ADHD, anxiety scores; (2) for site, age, sex, medication, IQ, handedness; (3) for site, age, sex, medication, IQ and handedness along with ADHD and anxiety scores. Abbreviations: ICU, Inventory of Callous-Unemotional Traits; RA, reactive aggression; RPQ, Reactive-Proactive Aggression Scale; PA, proactive aggression.


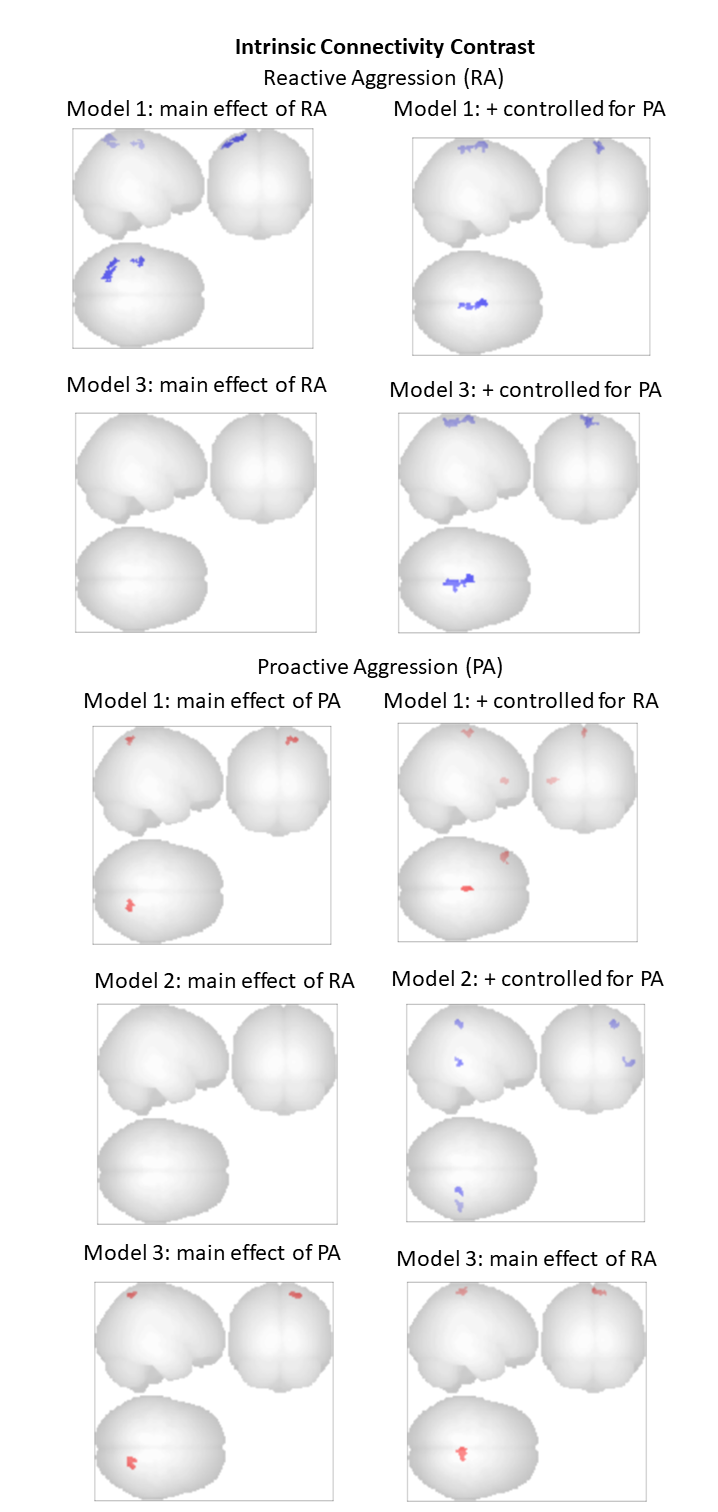


Supplementary Figure 2. ICC results of voxelwise regression analyses adjusted for RA/PA scores (RA for PA and vice versa). Model 1 - covariates: site, age, sex, medication, IQ, handedness. Model 2 - covariates: site, ADHD symptoms, anxiety symptoms. Model 3 - covariates: site, age, sex, medication, IQ, handedness, ADHD symptoms, anxiety symptoms. Left panel: results without the adjustment for PA/RA. Right panel: results with the adjustment for PA/RA (anatomical regions from the top down: 1. Right pre-central/post-central gyrus. 2. Right pre-central/post-central gyrus. 3. Left lateral frontal cortex, right supplementary motor area. 4. Postcentral gyrus, right middle/superior temporal gyrus. 5. Right Pre-central/post-central gyrus.


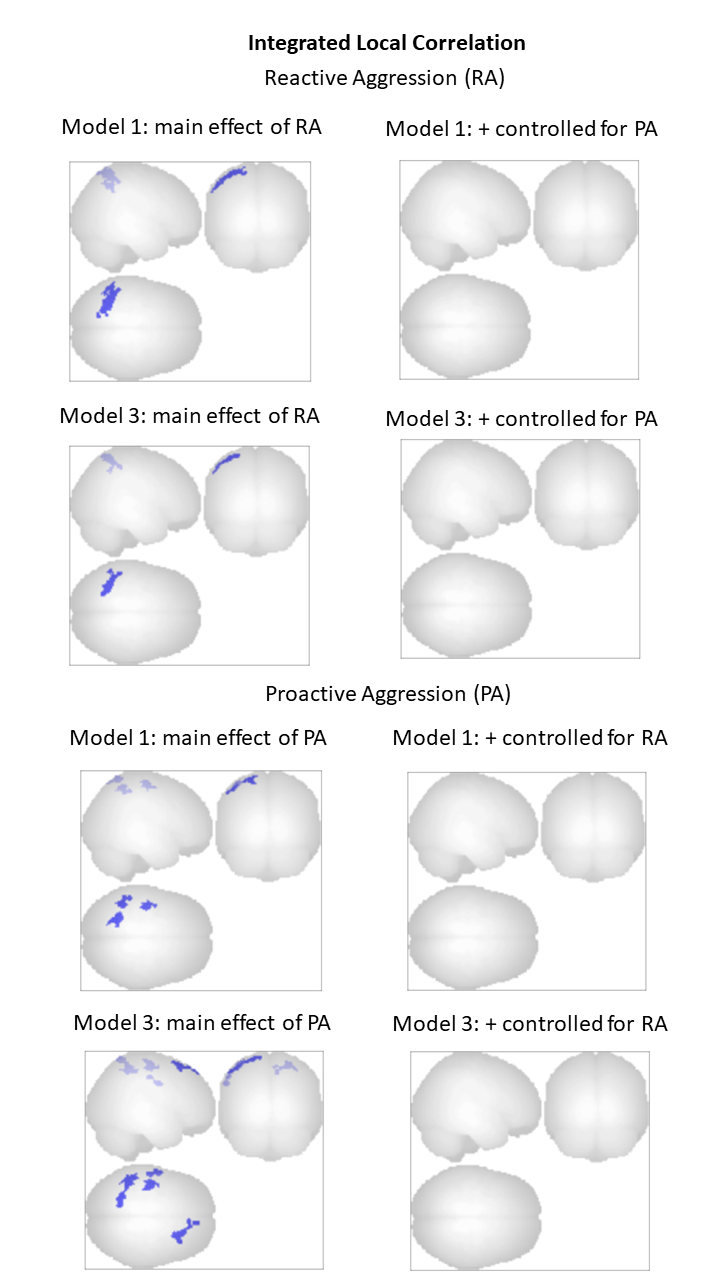


Supplementary Figure 3. ILC results of voxelwise regression analyses adjusted for RA/PA scores (RA for PA and vice versa). Model 1 - covariates: site, ADHD symptoms, anxiety symptoms. Model 2 - covariates: site, age, sex, medication, IQ, handedness. Model 3 - covariates: site, age, sex, medication, IQ, handedness, ADHD symptoms, anxiety symptoms. Left panel: results without the adjustment for PA/RA. Right panel: results with the adjustment for PA/RA.

**SUPPLEMENTARY TABLE 8.**

Clusters and coordinates derived from intrinsic connectivity contrast (ICC) results related to reactive, proactive, and general aggression when additionally controlled for callous-unemotional (CU) traits.

|  |  |  |  |  |  | MNI Coordinates | | |
| --- | --- | --- | --- | --- | --- | --- | --- | --- |
|  | Behavioral  Measure | Brain region | Hemisphere | Cluster size | *β*-value | *X* | *Y* | *Z* |
| **1** | RPQ – RA | *Negative Association*  -Superior Parietal/Lateral Occipital Cortex | L | 111 | -0.48 | -32 | -56 | 68 |
|  |  | Precentral/Postcentral Gyrus | L | 62 | -0.49 | -48 | -12 | 58 |
|  | RPQ- PA | *Positive Association*  -Superior Parietal Lobe/Lateral Occipital cortex | R | 80 | 0.30 | 26 | -56 | 62 |
|  | RPQ-total | *Negative Association*  - Pre-central/Post-central Gyrus | L | 131 | -0.22 | -48 | -12 | 58 |
|  |  | -Superior Parietal Lobe/Lateral Occipital cortex | L | 72 | -0.22 | -42 | -56 | 60 |
| **2** | RPQ – RA | No significant results |  |  |  |  |  |  |
|  | RPQ- PA | No significant results |  |  |  |  |  |  |
|  | RPQ- total | No significant results |  |  |  |  |  |  |
| **3** | RPQ – RA | No significant results |  |  |  |  |  |  |
|  | RPQ- PA | *Positive Association*  -Superior Parietal Lobe/Lateral Occipital cortex | L | 68 | 0.30 | -32 | -60 | 50 |
|  |  | -Superior Parietal Lobe/Lateral Occipital Cortex | R | 77 | 0.34 | 26 | -56 | 62 |
|  | RPQ- total | *Negative Association*  -Precentral/Postcentral Gyrus | L | 78 | -0.24 | -48 | -12 | 58 |

*Note:* The statistical threshold for the reported results is *p* < 0.001, false discovery rate (FDR) cluster-level corrected (*p* < 0.05) for multiple comparisons. Β-values represent *z*-standardized correlation coefficients. The following corrections were applied: (1) for site, ADHD, anxiety scores; (2) for site, age, sex, medication, IQ, handedness; (3) for site, age, sex, medication, IQ and handedness along with ADHD and anxiety scores. Abbreviations: RA, reactive aggression; RPQ, Reactive-Proactive Aggression Scale; PA, proactive aggression.

**SUPPLEMENTARY TABLE 9.**

Clusters and coordinates derived from integrated local correlation (ILC) results related to reactive, proactive, and general aggression when additionally controlled for callous-unemotional (CU) traits.

|  |  |  |  |  |  | MNI Coordinates | | |
| --- | --- | --- | --- | --- | --- | --- | --- | --- |
|  | Behavioral  Measure | Brain region | Hemisphere | Cluster size | *β*-value | *X* | *Y* | *Z* |
| **1** | RPQ – RA | *Negative Association*  -Superior Parietal Lobe/ Lateral Occipital Cortex/ Supramarginal Gyrus/ Angular Gyrus | L | 271 | -0.33 | -32 | -54 | 68 |
|  | RPQ- PA | *Negative Association*  -Precentral/Postcentral Gyrus | L | 136 | -0.29 | -42 | -16 | 62 |
|  | RPQ-total | *Negative Association*  *-*Superior Parietal Lobe/Lateral Occipital Cortex/ Supramarginal Gyrus/ Angular Gyrus | L | 258 | -0.15 | -30 | -56 | 68 |
|  |  | -Precentral/Postcentral Gyrus | L | 117 | -0.18 | -42 | -16 | 62 |
| **2** | RPQ – RA | No significant results |  |  |  |  |  |  |
|  | RPQ- PA | *Negative Association*  Precentral/Postcentral Gyrus | L | 115 | -0.28 | -40 | -16 | 62 |
|  | RPQ- total | No significant results |  |  |  |  |  |  |
| **3** | RPQ – RA | *Negative Association*  - Superior Parietal Lobe/Lateral Occipital Cortex/ Supramarginal Gyrus | L | 188 | -0.36 | -38 | -50 | 64 |
|  | RPQ- PA | *Negative Association*  -Precentral/Postcentral Gyrus | L | 250 | -0.32 | -42 | -16 | 62 |
|  |  | -Superior Parietal Lobe/Lateral Occipital Cortex/ Supramarginal Gyrus | L | 143 | -0.25 | -40 | -50 | 62 |
|  |  | - Superior Frontal Gyrus/ Middle Frontal Gyrus | R | 119 | -0.25 | 24 | 28 | 60 |
|  |  | - Superior Frontal Gyrus/ Middle Frontal Gyrus | L | 78 | -0.31 | -36 | 22 | 52 |
|  |  |  |  |  |  |  |  |  |
|  |  |  |  |  |  |  |  |  |
| **Supplementary Table 9 continued** | | | | | | | | |
|  | RPQ- total | *Negative Association*  *-*Superior Parietal Lobe/Lateral Occipital Cortex/ Supramarginal Gyrus/ Angular Gyrus | L | 242 | -0.20 | -40 | -50 | 62 |
|  |  |  |  |  |  |  |  |  |
|  |  | -Precentral/Postcentral Gyrus | L | 241 | -0.17 | -42 | -16 | 62 |
|  |  | -Superior Frontal Gyrus/ Middle Frontal Gyrus | R | 98 | -0.15 | 24 | 28 | 60 |

*Note:* The statistical threshold for the reported results is *p* < 0.001, false discovery rate (FDR) cluster-level corrected (*p* < 0.05) for multiple comparisons. Β-values represent *z*-standardized correlation coefficients. The following corrections were applied: (1) for site, ADHD, anxiety scores; (2) for site, age, sex, medication, IQ, handedness; (3) for site, age, sex, medication, IQ and handedness along with ADHD and anxiety scores. Abbreviations: RA, reactive aggression; RPQ, Reactive-Proactive Aggression Scale; PA, proactive aggression.

**
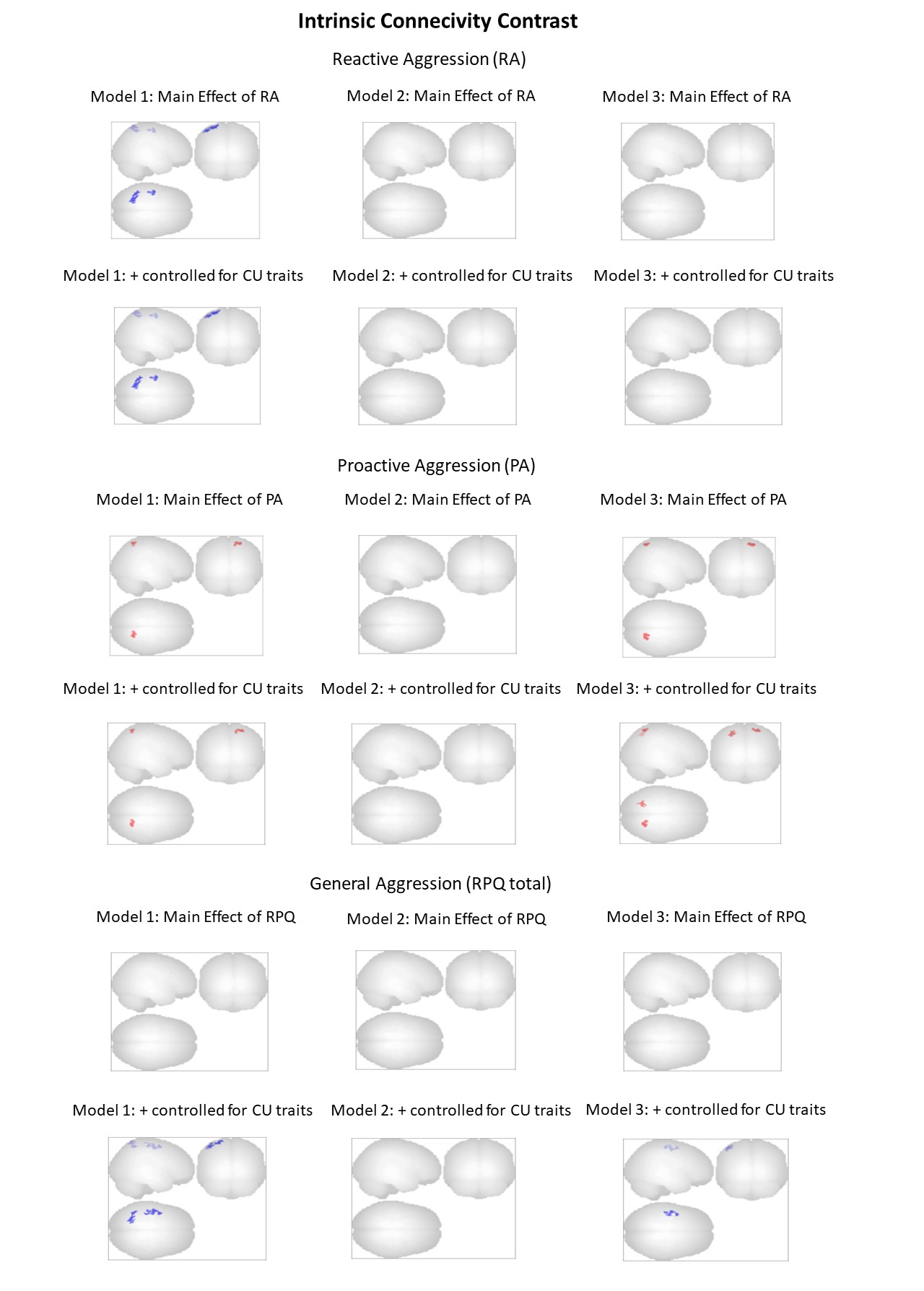
**

**Supplementary Figure 4.** ICC results for reactive, proactive and general aggression when controlled for callous-unemotional (CU) traits. Model 1 - covariates: site, ADHD symptoms, anxiety symptoms. Model 2 - covariates: site, age, sex, medication, IQ, handedness. Model 3 - covariates: site, age, sex, medication, IQ, handedness, ADHD symptoms, anxiety symptoms.


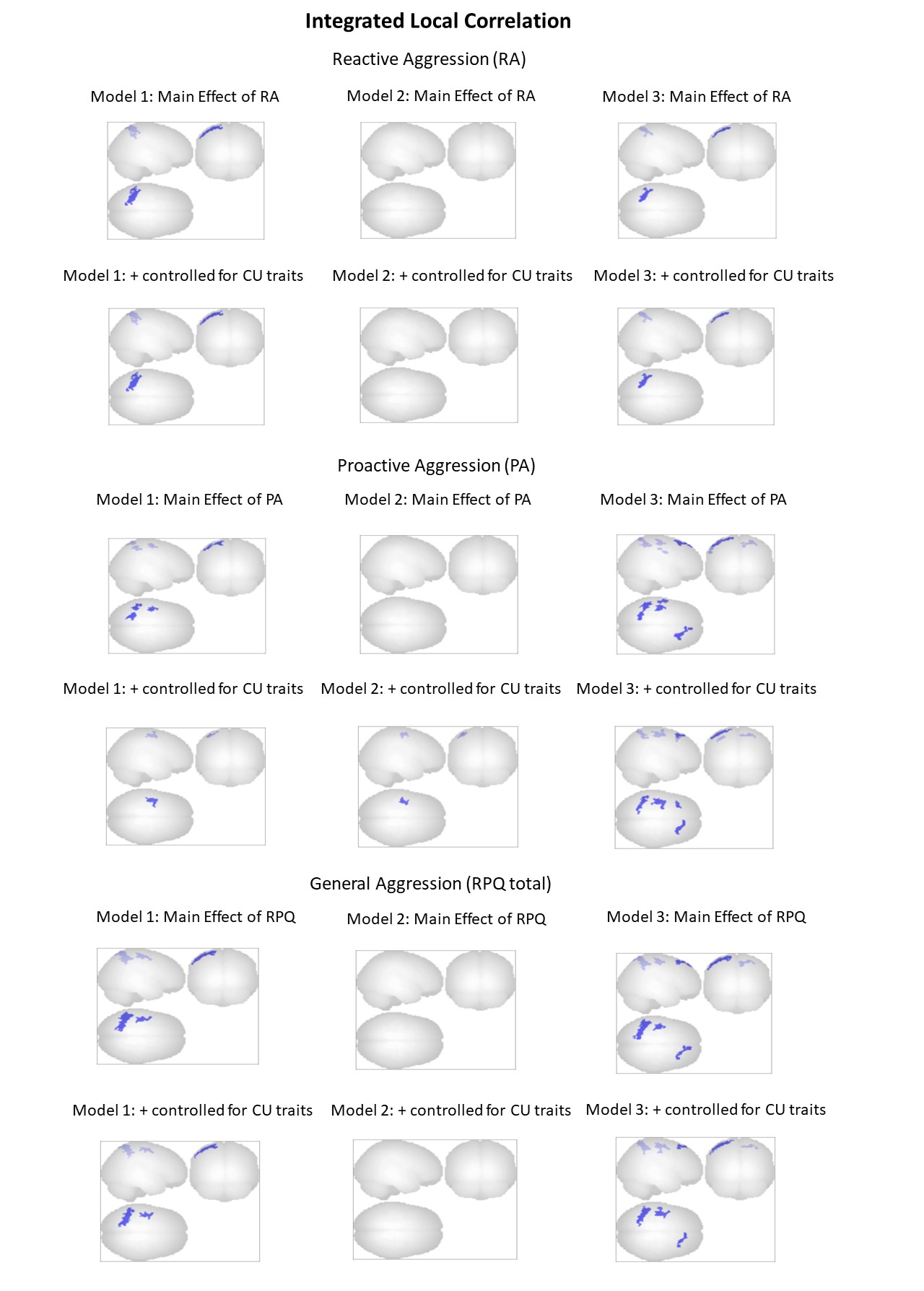
**Supplementary Figure 5.** ILC results for reactive, proactive and general aggression when controlled for callous-unemotional (CU) traits. Model 1 - covariates: site, ADHD symptoms, anxiety symptoms. Model 2 - covariates: site, age, sex, medication, IQ, handedness. Model 3 - covariates: site, age, sex, medication, IQ, handedness, ADHD symptoms, anxiety symptoms.

**Supplemental References**

Achenbach, T.M., 1991. Integrative guide for the 1991 CBCL/4-18, YSR, and TRF profiles. Burlington, VT, USA.

Allen, E.A., Erhardt, E.B., Damaraju, E., Gruner, W., Segall, J.M., Silva, R.F., Havlicek, M., Rachakonda, S., Fries, J., Kalyanam, R., Michael, A.M., Caprihan, A., Turner, J.A., Eichele, T., Adelsheim, S., Bryan, A.D., Bustillo, J., Clark, V.P., Ewing, S.W.F., Filbey, F., Ford, C.C., Hutchison, K., Jung, R.E., Kiehl, K.A., Kodituwakku, P., Komesu, Y.M., Mayer, A.R., Pearlson, G.D., Phillips, J.P., Sadek, J.R., Stevens, M., Teuscher, U., Thoma, R.J., Calhoun, V.D., 2011. A baseline for the multivariate comparison of resting-state networks. Front. Syst. Neurosci. https://doi.org/10.3389/fnsys.2011.00002

Cools, R., Clark, L., Owen, A.M., Robbins, T.W., 2002. Defining the Neural Mechanisms of Probabilistic Reversal Learning Using Event-Related Functional Magnetic Resonance Imaging. J. Neurosci. 22. https://doi.org/10.1523/jneurosci.22-11-04563.2002

Finger, E.C., Marsh, A.A., Blair, K.S., Reid, M.E., Sims, C., Ng, P., Pine, D.S., Blair, R.J.R., 2011. Disrupted reinforcement signaling in the orbitofrontal cortex and caudate in youths with conduct disorder or oppositional defiant disorder and a high level of psychopathic traits. Am. J. Psychiatry 168. https://doi.org/10.1176/appi.ajp.2010.10010129

Frick, P.J., 2004. The inventory of callous-unemotional traits. Unpublished rating scale.

Goodman, R., 1997. The strengths and difficulties questionnaire: A research note. J. Child Psychol. Psychiatry Allied Discip. 38. https://doi.org/10.1111/j.1469-7610.1997.tb01545.x

Kaufman, J., Birmaher, B., Brent, D., Rao, U., Flynn, C., Moreci, P., Williamson, D., Ryan, N., 1997. Schedule for affective disorders and schizophrenia for school-age children-present and lifetime version (K-SADS-PL): Initial reliability and validity data. J. Am. Acad. Child Adolesc. Psychiatry 36. https://doi.org/10.1097/00004583-199707000-00021

Kay, S.R., Wolkenfeld, F., Murrill, L.M., 1988. Profiles of aggression among psychiatric patients: I. Nature and prevalence. J. Nerv. Ment. Dis. 176. https://doi.org/10.1097/00005053-198809000-00007

Liu, H., Stufflebeam, S.M., Sepulcre, J., Hedden, T., Buckner, R.L., 2009. Evidence from intrinsic activity that asymmetry of the human brain is controlled by multiple factors. Proc. Natl. Acad. Sci. U. S. A. 106. https://doi.org/10.1073/pnas.0908073106

Mak, L.E., Minuzzi, L., MacQueen, G., Hall, G., Kennedy, S.H., Milev, R., 2017. The Default Mode Network in Healthy Individuals: A Systematic Review and Meta-Analysis. Brain Connect. https://doi.org/10.1089/brain.2016.0438

Muehlhan, M., Lueken, U., Wittchen, H.-U., Kirschbaum, C., 2011. The scanner as a stressor: evidence from subjective and neuroendocrine stress parameters in the time course of a functional magnetic resonance imaging session. Int J Psychophysiol. 79, 118–26.

Ormel, J., Oldehinkel, A.J., Sijtsema, J., Van Oort, F., Raven, D., Veenstra, R., Vollebergh, W.A.M., Verhulst, F.C., 2012. The TRacking Adolescents’ Individual Lives Survey (TRAILS): Design, current status, and selected findings. J. Am. Acad. Child Adolesc. Psychiatry 51. https://doi.org/10.1016/j.jaac.2012.08.004

Petersen, A.C., Crockett, L., Richards, M., Boxer, A., 1988. A self-report measure of pubertal status: Reliability, validity, and initial norms. J. Youth Adolesc. 17. https://doi.org/10.1007/BF01537962

Raine, A., Dodge, K., Loeber, R., Gatzke-Kopp, L., Lynam, D., Reynolds, C., Stouthamer-Loeber, M., Liu, J., 2006. The reactive-proactive aggression questionnaire: Differential correlates of reactive and proactive aggression in adolescent boys. Aggress. Behav. 32. https://doi.org/10.1002/ab.20115

Robbins, T.W., James, M., Owen, A.M., Sahakian, B.J., McInnes, L., Rabbitt, P., 1994. Cambridge neuropsychological test automated battery (CANTAB): A factor analytic study of a large sample of normal elderly volunteers. Dementia. https://doi.org/10.1159/000106735

Rubia, K., Halari, R., Mohammad, A.M., Taylor, E., Brammer, M., 2011. Methylphenidate normalizes frontocingulate underactivation during error processing in attention-deficit/hyperactivity disorder. Biol. Psychiatry 70. https://doi.org/10.1016/j.biopsych.2011.04.018

Sherman, L.E., Rudie, J.D., Pfeifer, J.H., Masten, C.L., McNealy, K., Dapretto, M., 2014. Development of the Default Mode and Central Executive Networks across early adolescence: A longitudinal study. Dev. Cogn. Neurosci. 10. https://doi.org/10.1016/j.dcn.2014.08.002

Slot, N., de Castro, B., Duivenvoorden, Y., 2000. WAS-lijst, eerste afname, versie 1.0. Paedologisch Instituut, Duivendrecht.

Swanson, J.M., 1992. School-based assessments and interventions for ADD students. KC Publishing.

Via, E., Cardoner, N., Pujol, J., Alonso, P., López-Solà, M., Real, E., Contreras-Rodríguez, O., Deus, J., Segalàs, C., Menchón, J.M., Soriano-Mas, C., Harrison, B.J., 2014. Amygdala activation and symptom dimensions in obsessive-compulsive disorder. Br. J. Psychiatry 204. https://doi.org/10.1192/bjp.bp.112.123364

Wechsler, D., 2003. Wechsler Intelligence Scale for Children-(WISC-IV Australian). Pearson Clinical and Talent Assessment, Sydney.
